# The SMARCA4^R1157W^ mutation facilitates chromatin remodeling and confers PRMT1/SMARCA4 inhibitors sensitivity in colorectal cancer

**DOI:** 10.1038/s41698-023-00367-y

**Published:** 2023-03-15

**Authors:** Xiangwei Zeng, Bing Yao, Jianpeng Liu, Guan-Wen Gong, Ming Liu, Jiahuang Li, Hua-Feng Pan, Qixiang Li, Dongjun Yang, Peifen Lu, Dongliang Wu, Peipei Xu, Bing Chen, Panhai Chen, Ming Zhang, Ke Zen, Jian Jing, David C. S. Huang, Dijun Chen, Zhi-Wei Jiang, Quan Zhao

**Affiliations:** 1 The State Key Laboratory of Pharmaceutical Biotechnology, Department of Hematology, the Affiliated Drum Tower Hospital of Nanjing University Medical School, China–Australia Institute of Translational Medicine, School of Life Sciences, Nanjing University, Nanjing, China; 2 Department of Medical Genetics, Nanjing Medical University, Nanjing, China; 3 Department of General Surgery, the Affiliated Hospital of Nanjing University of Chinese Medicine, Jiangsu Province Hospital of Chinese Medicine, Nanjing, China; 4 China–Australia Institute of Translational Medicine Co. Ltd, Nanjing, China; 5 The Walter and Eliza Hall Institute of Medical Research, Department of Medical Biology, University of Melbourne, Melbourne, VIC Australia

**Keywords:** Epigenetics, Colorectal cancer

## Abstract

Genomic studies have demonstrated a high frequency of genetic alterations in components of the SWI/SNF complex including the core subunit SMARCA4. However, the mechanisms of tumorigenesis driven by SMARCA4 mutations, particularly in colorectal cancer (CRC), remain largely unknown. In this study, we identified a specific, hotspot mutation in *SMARCA4* (c. 3721C>T) which results in a conversion from arginine to tryptophan at residue 1157 (R1157W) in human CRC tissues associated with higher-grade tumors and controls CRC progression. Mechanistically, we found that the SMARCA4^R1157W^ mutation facilitated its recruitment to PRMT1-mediated H4R3me2a (asymmetric dimethylation of Arg 3 in histone H4) and enhanced the ATPase activity of SWI/SNF complex to remodel chromatin in CRC cells. We further showed that the SMARCA4^R1157W^ mutant reinforced the transcriptional expression of *EGFR* and *TNS4* to promote the proliferation of CRC cells and patient-derived tumor organoids. Importantly, we demonstrated that SMARCA4^R1157W^ CRC cells and mutant cell-derived xenografts were more sensitive to the combined inhibition of PRMT1 and SMARCA4 which act synergistically to suppress cell proliferation. Together, our findings show that SMARCA4-R1157W is a critical activating mutation, which accelerates CRC progression through facilitating chromatin recruitment and remodeling. Our results suggest a potential precision therapeutic strategy for the treatment of CRC patients carrying the SMARCA4^R1157W^ mutation.

## Introduction

Colorectal cancer (CRC) is the third most frequent cancer and the second most common cause of cancer-related mortality worldwide^1^. The global burden of CRC is expected to increase by 60% in the coming years^1^. This increase is associated with a deleterious lifestyle, obesity, and lack of exercise, which causes the accumulation of genetic and epigenetic alterations in colorectal epithelial cells, thereby driving the initiation and progression of CRC^1,2^. Over the past decade, advances have been made in understanding CRC epigenetics, particularly regarding aberrant regulation of DNA methylation, histone modification, and chromatin remodeling and chromosome architecture^3–5^. Progress in this field in recent years has advanced our knowledge and ability to tackle these epigenetic alterations for the prevention and treatment of CRC.

In eukaryotic cells, the fundamental unit of chromatin is the nucleosome, which is formed by 147 bp of DNA wrapped around an octamer of the four core histones (H2A, H2B, H3, and H4)^6^. Biochemical and genetic evidence suggests that nucleosomes normally inhibit transcription by blocking access of binding factors to their cognate DNA binding sites^7^. Thus, transcription usually requires factors, such as chromatin remodelers, to overcome the formidable obstacles caused by nucleosomes. The SWItch/Sucrose NonFermentable (SWI/SNF) complex is an ATP-dependent chromatin remodeling complex composed of approximately 15 protein subunits, which plays an important role in the regulation of gene transcription, cell cycle, DNA replication, DNA repair, and stress response in eukaryotes^8,9^. The complex dynamically alters the structure of chromatin by disrupting the contact between octamer histones and DNA, thereby regulating gene transcription^10^. SMARCA4 (SWI/SNF-related matrix-associated actin-dependent regulator of chromatin subfamily A member 4, also known as Brahma-related gene 1, Brg1) is one of two mutually exclusive catalytic subunits in the SWI/SNF complex (the other is SMARCA2). SMARCA4 utilizes energy derived from ATP hydrolysis to remodel chromatin or change chromosome structure^8^. Studies have shown that SMARCA4 has either tumor-suppressing or tumor-promoting activities in a cancer context-specific manner^11^. Specifically, in the skin, blood (lymphoma), and ovarian and lung cancers, SMARCA4 functions as a tumor suppressor^12–15^. In contrast, other studies have reported tumorigenic effects of SMARCA4 on the development of prostate cancer, gastric cancer, and breast cancer^16–18^. SMARCA4 also plays diverse roles in CRC. For example, Liu et al. reported that SMARCA4 reduced colon inflammation and colorectal tumorigenesis through autophagy-dependent oxidative stress sequestration^19^; other reports revealed tumor-promoting effects of SMARCA4 in CRC^20–22^. Thus, there are some discrepancies regarding the functions of SMARCA4 with respect to tumor initiation and progression in the gut. In our previous studies, we found that PRMT1-mediated H4R3me2a (ω-N^G^, N^G^-asymmetric dimethylation of the guanidine nitrogen of Arg 3 in histone H4) recruits the wild-type SMARCA4 to promote colorectal cancer progression by enhancing EGFR signaling, providing another example of an oncogenic role of SMARCA4 in CRC^23^.

Notably, genes encoding subunits of SWI/SNF are mutated in more than 20% of all human cancers, making the SWI/SNF complex the most commonly mutated chromatin modulator in humans^24,25^. SMARCA4 is a commonly mutated subunit, with a mutation frequency of 8% in lung adenocarcinoma^26^. SMARCA4-inactivating mutations (loss-of-function) have been identified as driver mutations in various cancers, including lung, ovarian, rhabdoid, and thoracic tumors^27–29^. However, to date, little is known about the functional role of SMARCA4 mutations in human CRC.

Here, we identified a critical activating mutation in SMARCA4 (R1157W) that increased the binding capability of SMARCA4 to PRMT1-mediated H4R3me2a and enhanced the ATPase activity and chromatin remodeling of the SWI/SNF complex, leading to the higher transcriptional output of *EGFR* and *TNS4* to accelerate the progression of CRC. We showed that combined inhibition of PRMT1 and SMARCA4 synergistically suppressed the growth of SMARCA4^R1157W^ mutant CRC cells in vitro and in vivo. These findings may provide a potential personalized therapeutic strategy for CRC patients with the SMARCA4^R1157W^ mutation.

## Results

### Identification of SMARCA4^R1157W^ specific mutation in CRC

**Fig. 1:**
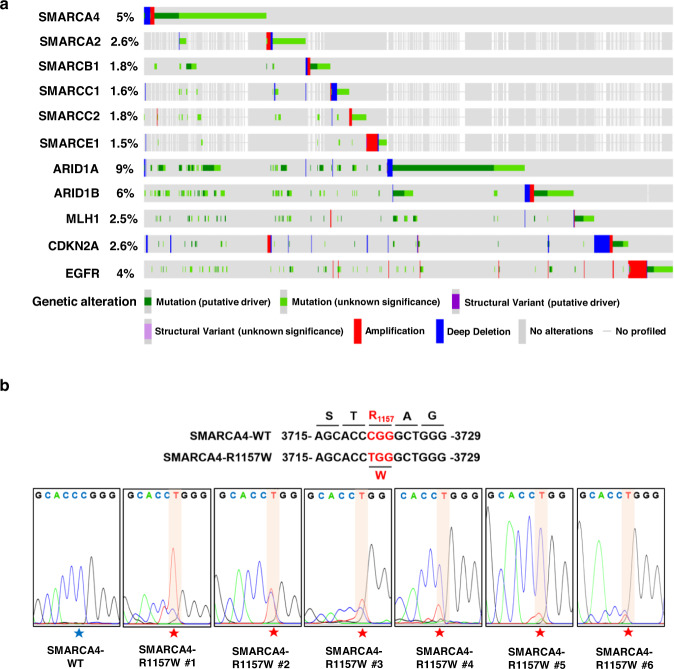
Identification of specific mutations of *SMARCA4* in CRC patients. **a** Oncoprint plot showing the genomic profiles of *SMARCA4*, *SMARCA2*, *SMARCB1*, *SMARCC1*, *SMARCC2*, *SMARCE1*, *ARID1A*, *ARID1B*, and other CRC relevant genes, data compiled from the cBioPortal based on original data from TCGA CRC project. **b** Detection of the *SMARCA4* gene showing the c. 3721C>T (p. Arg1157Trp) mutation in tumor samples of CRC patients using DNA sequencing.

To identify the key genetic mutations of SWI/SNF complex subunits associated with CRC, we analyzed genetic alterations of *SMARCA4 (BRG1)*, *SMARCA2 (BRM)*, *SMARCB1 (BAF47 or SNF5)*, *SMARCC1 (BAF155)*, *SMARCC2 (BAF170)*, *SMARCE1 (BAF57)*, *ARID1A (BAF250A)*, *ARID1B (BAF250B)*, and other common oncogenes (*MLH1*, *CDKN2A*, and *EGFR*) in CRC from the cBioPortal database^30^. We found that the mutation frequency of SMARCA4 was as high as 5% (Fig. 1a). These genetic alterations are primarily composed of single nucleotide missense mutations, amplifications, or deletions. Further data analysis showed that the *SMARCA4* harbored potential site-specific mutations in CRC, including R1157W, R1157Q, and R1243Q, which differed from the distribution of SMARCA4 mutations among other high-incidence and high-mortality cancers (lung cancer, liver cancer, and gastric cancer) (Supplementary Fig. 1). Interestingly, these specific mutation sites are in the ATPase domain (aa728–1388) based on the structural composition of SMARCA4 (Supplementary Fig. 1). To investigate the clinical significance of the SMARCA4 specific hotspot mutations in CRC patients, we collected 64 clinical samples from patients with CRC, and performed mutation screening for SMARCA4-R1157W, R1157Q, and R1243Q using direct DNA sequencing. We obtained 6 cases of R1157W mutation, although we did not observe R1157Q or R1243Q mutations in these collected samples (Fig. 1b). The conversion of the arginine codon (CGG) to tryptophan codon (TGG) (p. Arg1157Trp) was due to the substitution of C to T at nucleotide 3721 (c. 3721C>T) of SMARCA4 (Fig. 1b). Of note, six cases of the R1157W mutation were all heterozygous. In addition, the analysis of pathological information from these clinical samples showed that the SMARCA4-R1157W (SMARCA4^R1157W^) mutation was associated with higher-grade CRC (Supplementary Table 1), suggesting greater malignancy in patients with SMARCA4^R1157W^ mutation than in patients with wild-type SMARCA4.

### SMARCA4-R1157W mutant promotes CRC cell proliferation

**Fig. 2:**
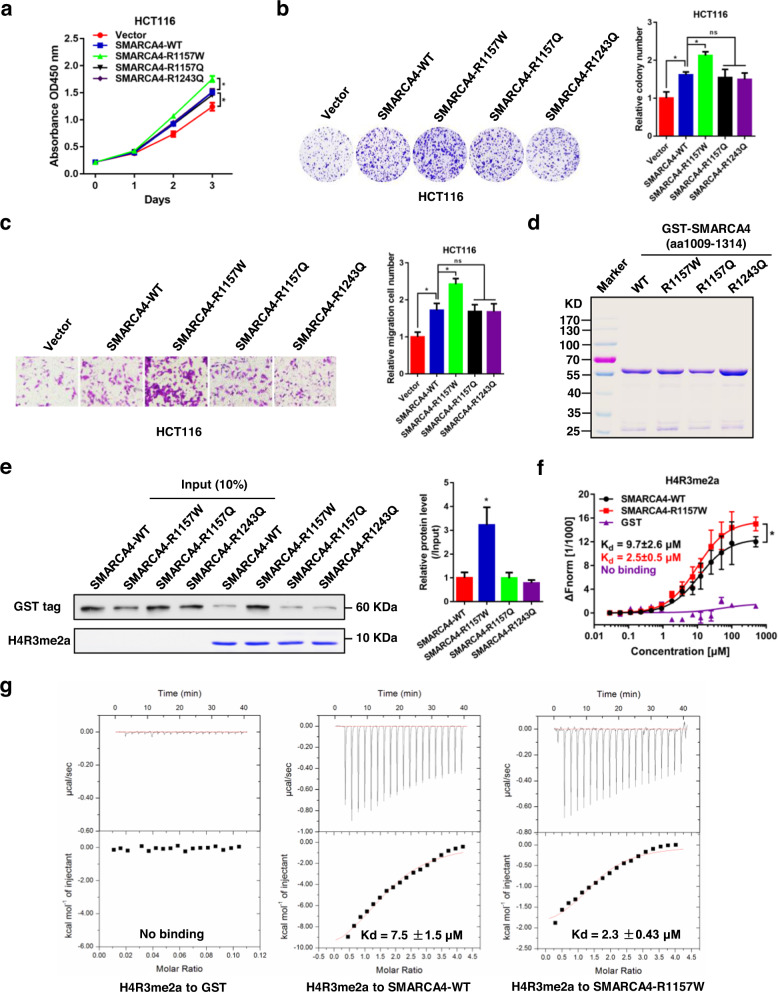
The SMARCA4-R1157W mutation enhances the binding to H4R3me2a and promotes CRC cell proliferation. **a** Proliferation of HCT116 cells transfected with SMARCA4 overexpression plasmids (SMARCA4 wild-type, R1157W, R1157Q, or R1243Q) or vector plasmids. Values at the indicated time points represent mean ± SD from three independent tests. *P < 0.05. **b** Colony formation assay of HCT116 cells transfected with SMARCA4 overexpression plasmids as in (**a**). Left, representative images; Right, group data. *P < 0.05. **c** Migration assays of HCT116 cells transfected with SMARCA4 overexpression plasmids as in (a). The numbers of migrated cells were quantified by counting the number of cells in entire fields at ×200 magnification. Left, representative images; Right, group data. *P < 0.05. **d** Coomassie blue staining of wild-type and mutant GST-labeled SMARCA4 fragment (aa1009–1314) proteins. **e** Peptide pull-down assay to detect the interactions between H4R3me2a peptide and wild-type and mutant GST-labeled SMARCA4 fragment proteins (left, top panel). Coomassie staining shows equivalent loading of the H4R3me2a peptide (left, bottom panel). Quantitative analysis of the peptide pull-down assay (right panel). *P < 0.05. **f**, **g** MST assay (**f**) and ITC assay (**g**) to detect the direct interactions between H4R3me2a peptide and GST-labeled SMARCA4 protein fragments (wild-type and mutant). *P < 0.05. In the graphs, the error bars are the standard deviation of the mean.

To investigate the potential effects of SMARCA4 wild-type (WT), R1157W, R1157Q, or R1243Q mutants on the growth of CRC cells, we transfected SMARCA4-WT, -R1157W, -R1157Q, or -R1243Q mutant overexpression plasmids into HCT116 and SW620 CRC cells. We examined cell growth by CCK-8, colony formation, and transwell migration assays. Overexpression of SMARCA4-WT significantly increased proliferation and colony formation capabilities of HCT116 and SW620 cells compared to vector controls (Fig. 2a, b; Supplementary Fig. 2a, b). Interestingly, proliferation and colony formation seen in SMARCA4-R1157W cells were significantly greater than seen in SMARCA4-WT cells, whereas proliferation and colony formation seen in SMARCA4-R1157Q and R1243Q cells were similar to proliferation and colony formation seen in SMARCA4-WT cells (Fig. 2a, b; Supplementary Fig. 2a, b). The results of the transwell migration experiment also showed patterns similar to the changes observed in cell proliferation and clone formation assays (Fig. 2c; Supplementary Fig. 2c).

### SMARCA4-R1157W mutation confers high H4R3me2a binding capability

Our previous studies have indicated that SMARCA4 could bind the PRMT1-mediated histone H4R3me2a modification and enhance EGFR signaling^23^. Therefore, we wondered whether the SMARCA4 mutations could affect the binding capacity of SMARCA4 to H4R3me2a. To test this possibility, we first purified recombinant GST-fusion wild-type and SMARCA4 mutant fragments (aa1009–1314) (Fig. 2d). We then performed peptide pulldown assays with a C-terminal biotin-labeled histone H4 peptide in which the Arg3 residue was asymmetrically dimethylated (H4R3me2a). We found that the SMARCA4-R1157W mutation significantly enhanced binding to the H4R3me2a peptide compared to binding to wild-type SMARCA4 protein, whereas the R1157Q or R1243Q mutant proteins provided no enhancement (Fig. 2e). In addition, we expressed wildtype and mutant SWI/SNF complex (assembled with SMARCA4-WT or -R1157W/R1157Q/R1243Q mutant) and performed peptide pulldown assays with the complexes. We found that the SWI/SNF complex containing SMARCA4-R1157W mutant significantly increased the binding to the H4R3me2a peptide, but not the SMARCA4-R1157Q/R1243Q SWI/SNF complex compared with SMARCA4-WT SWI/SNF complex (Supplementary Fig. 2d). These results imply that the SMARCA4-R1157W mutant may have increased electrostatic attraction and/or hydrogen bonding capacity over the R1157Q or R1243Q mutant proteins relevant to binding the H4R3me2a peptide (Supplementary Fig. 2e). The change in binding energy suggests that the affinity of the SMARCA4-R1157W mutant protein for the H4R3me2a peptide was significantly enhanced compared with that of the SMACA4-WT protein (Supplementary Fig. 2e). We further assessed the binding capability of SMARCA4-WT or R1157W mutant proteins to the H4R3me2a peptide by microscale thermophoresis (MST) assays. For the binding of wild-type or mutant SMARCA4-R1157W proteins to the H4R3me2a peptide, the data were fitted to single-site-binding models with dissociation constants (*K*_d_) of 9.7 ± 2.6 or 2.5 ± 0.5 μM, respectively (Fig. 2f, *P* < 0.05). No binding of the GST control protein to the H4R3me2a peptide was observed (Fig. 2f). Consistent with the pulldown and MST results, using isothermal titration calorimetry (ITC) assays, we further confirmed that the R1157W mutation enhanced the binding ability of the SMARCA4 protein to the H4R3me2a peptide. The *K*_d_ values of the binding of wild-type or R1157W mutant with the H4R3me2a peptide were 7.5 ± 1.5 or 2.3 ± 0.43 μM, respectively (Fig. 2g, *P* < 0.05). These results indicate that the binding affinity of the SMARCA4 protein to the H4R3me2a peptide was significantly elevated in the presence of the R1157W mutation.

### SMARCA4-R1157W mutation reinforces *EGFR* and *TNS4* expression to accelerate CRC cell proliferation

**Fig. 3:**
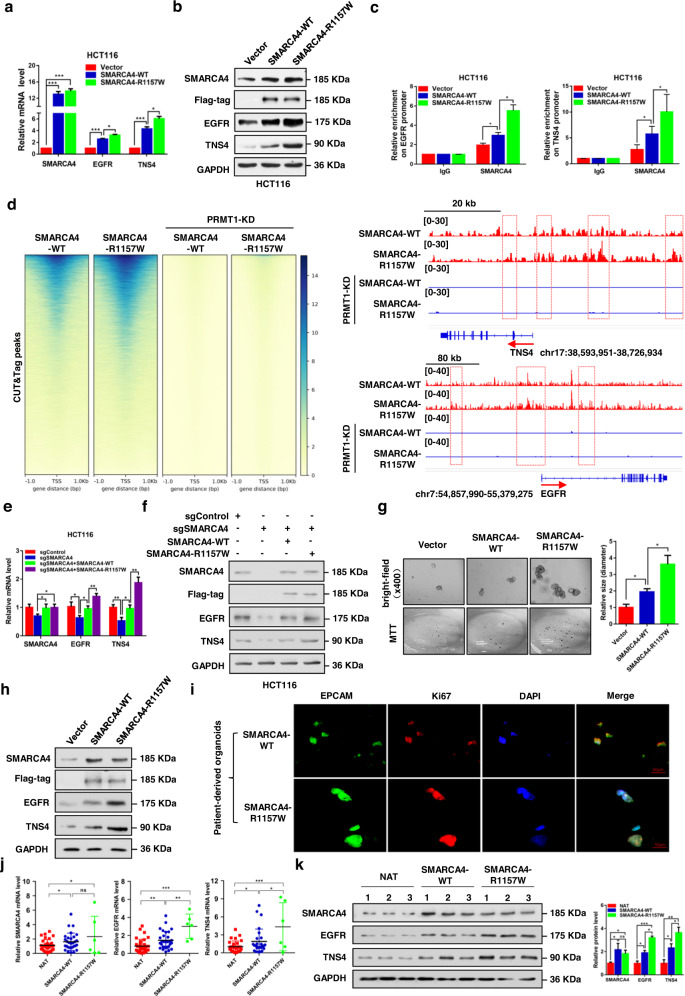
The SMARCA4-R1157W mutant promotes the growth of CRC cells and CRC patient-derived organoids. **a** Quantitative real-time PCR analysis of *SMARCA4*, *EGFR*, and *TNS4* mRNA levels normalized to GAPDH in HCT116 cells transfected with SMARCA4-WT and R1157W mutant overexpression plasmids. *P < 0.05, ***P < 0.001. **b** Western blot analysis of indicated proteins in HCT116 cells transfected with SMARCA4-WT and R1157W mutant overexpression plasmids. GAPDH served as a loading control. **c** ChIP analysis of SMARCA4-WT and SMARCA4-R1157W binding to the *EGFR* and *TNS4* promoter in HCT116 cells. *P < 0.05. **d** Heatmaps of CUT&Tag signals show SMARCA4 read density around TSS in SMARCA4-WT or SMARCA4-R1157W HCT116 cells, and these cells with PRMT1 knockdown within a ±1 kb window (left panels). CUT & Tag profiling tracks of SMARCA4 in the vicinity of *TNS4* and *EGFR* loci in SMARCA4-R1157W versus SMARCA4-WT HCT116 cells, and these cells with PRMT1 knockdown. Track height is normalized to a relative number of mapped reads (right panels). **e** Quantitative real-time PCR analysis of *SMARCA4*, *EGFR*, and *TNS4* mRNA levels normalized to GAPDH in SMARCA4-KO HCT116 cells transfected with SMARCA4-WT or SMARCA4-R1157W mutant plasmids. *P < 0.05, **P < 0.01. **f** Western blot analysis of indicated proteins in SMARCA4-KO HCT116 cells transfected with SMARCA4-WT or SMARCA4-R1157W mutant plasmids. GAPDH served as a loading control. **g** CRC patient-derived organoids using 3D culture systems were generated, and SMARCA4-WT or SMARCA4-R1157W plasmids were transfected into the CRC organoids. (left) Representative microscopic images (top), MTT staining (bottom) on day 9. (right) Quantitative analyses of organoid size at day 9. *P < 0.05. **h** Western blot analysis of indicated proteins in CRC organoids transfected with SMARCA4-WT or SMARCA4-R1157W plasmids. GAPDH served as a loading control. **i** Immunofluorescence staining of EPCAM (green), Ki67 (red), and DAPI (blue) in CRC patient-derived SMARCA-WT and SMARCA4-R1157W mutant organoids. **j** Quantitative real-time PCR analysis of *SMARCA4*, *EGFR,* and *TNS4* mRNA levels normalized to GAPDH in adjacent normal colon tissue controls (NAT) (*n* = 32), SMARCA4-WT (*n* = 30) or SMARCA4-R1157W mutant (*n* = 6) CRC human tissues. *P < 0.05, **P < 0.01, ***P < 0.001. **k** Western blot analysis of indicated proteins in three representative CRC human tissues (left panel): NAT, SMARCA4-WT, or SMARCA4-R1157W mutant. GAPDH was used as the loading control. Quantitative analysis of the western blot assay (right panel). *P < 0.05, **P < 0.01, ***P < 0.001. In the graphs, the error bars are the standard deviation of the mean.

Next, we assessed key downstream target genes which are regulated by SMARCA4-R1157W in CRC cells. Previously, we showed that *EGFR* and *TNS4* (also named CTEN which regulates EGFR protein levels through a posttranslational mechanism, and prolongs signaling by EGFR through reducing its ligand-induced degradation^31^) were major direct downstream transcriptional targets of SMARCA4 in colon cells regulated in a PRMT1 methyltransferase activity-dependent manner to promote CRC cell proliferation^23^. Here we used immunofluorescence staining experiments to show that the R1157W mutation in SMARCA4 did not alter its nuclear localization in CRC cells, suggesting that SMARCA4-R1157W has a similar traffic pathway as the wild-type (Supplementary Fig. 3a). Thus, we evaluated the effect of SMARCA4-R1157W on the expression of *EGFR* and *TNS4* genes. We found that overexpression of SMARCA4-WT in HCT116 or SW620 cells led to significantly increased mRNA and protein expression of *EGFR* and *TNS4* compared to the vector group (Fig. 3a, b; Supplementary Fig. 4a, b). Moreover, SMARCA4-R1157W resulted in even higher mRNA and protein expression levels of *EGFR* and *TNS4* compared to the SMARCA4-WT group (Fig. 3a, b; Supplementary Fig. 4a, b). Consistently, the ChIP analyses using an anti-SMARCA4 antibody showed that overexpression of SMARCA4-WT resulted in a significant enrichment of SMARCA4 at the promoters of *EGFR* and *TNS4* compared to the vector group in HCT116 cells or SW620 cells (Fig. 3c; Supplementary Fig. 4c). In addition, overexpression of SMARCA4-R1157W mutation led to a much greater enrichment of SMARCA4 at the promoters of *EGFR* and *TNS4* compared to SMARCA4-WT group (Fig. 3c; Supplementary Fig. 4c). Indeed, data from SMARCA4 CUT & Tag experiments showed that the SMARCA4-R1157W mutant displayed higher global enrichment at transcriptional start sites (TSSs) than SMARCA4-WT, and showed higher local enrichment at the promoters, transcriptional start sites and gene body regions of *TNS4* and *EGFR* than SMARCA4-WT (Fig. 3d), which was consistent with our ChIP results (Fig. 3c). To confirm the role of PRMT1-mediated H4R3me2a in facilitating SMARCA4 binding in CRC cells, we performed SMARCA4 CUT & Tag experiments in CRC cells with PRMT1 depletion. We found that PRMT1 loss resulted in the loss of SMARCA4 binding at TSSs globally, and the loss of SMARCA4 binding at gene regions of *TNS4* and *EGFR* in both SMARCA4-WT and SMARCA4-R1157W mutant cells, suggesting that H4R3me2a is critical for SMARCA4 binding to chromatin of CRC cells (Fig. 3d; Supplementary Fig. 4d).

To further determine the effect of SMARCA4-R1157W mutation on CRC cell growth, we established CRISPR-mediated SMARCA4 knockouts in HCT116 (Fig. 3f) and SW620 (Supplementary Fig. 4i) cell lines using specific guide RNA. We then stably overexpressed SMARCA4-WT or SMARCA4-R1157W in these knockout cells. We found that knockout of SMARCA4 significantly suppressed cell growth, colony formation, and migration ability of both HCT116 and SW620 cell lines and that the cells proliferated faster when rescued by SMARCA4-R1157W than when rescued by SMARCA4-WT (Supplementary Fig. 4e–g). Moreover, higher levels of *EGFR* and *TNS4* mRNA and protein were detected by qRT-PCR and western blot analysis in cells rescued by SMARCA4-R1157W than in cells rescued by SMARCA4-WT (Fig. 3e, f; Supplementary Fig. 4h, i). Consistently, we showed the binding of SMARCA4-R1157W to H4R3me2a was significantly increased compared to the SMARCA4-WT by coimmunoprecipitation experiments and a ChIP-reChIP strategy, in which chromatin immunoprecipitated with H4R3me2a antibody was re-immunoprecipitated with antibody to SMARCA4 (Supplementary Fig. 3b, c). In addition, we generated CRC patient-derived organoids using three-dimensional (3D) culture systems and transfected these organoids with SMARCA4 overexpression plasmids. We found that the numbers and average sizes of organoids following transfection with SMARCA4-R1157W were larger than those seen following transfection of SMARCA4-WT (Fig. 3g). As expected, the transfection of SMARCA4-R1157W in CRC organoids led to significantly higher expression levels of *EGFR* and *TNS4* genes (Fig. 3h). Furthermore, we successfully established a CRC patient organoid containing the SMARCA4-R1157W mutation. We found that SMARCA4-R1157W mutant organoids were not only significantly larger in size, but also showed increased EPCAM and Ki67 immunofluorescence staining compared to SMARCA4-WT organoids (Fig. 3i). The data indicate a proliferative advantage of SMARCA4-R1157W mutant organoids. More importantly, qRT–PCR and western blot analysis on tissues from CRC patients showed much higher EGFR and TNS4 expression in SMARCA4-R1157W mutant CRCs compared to SMARCA4-WT CRCs (Fig. 3j, k), consistent with the results of cellular experiments. Moreover, as six cases of R1157W mutation patients were all heterozygous (Fig. 1b), we have constructed the SMARCA4-R1157W heterozygous (HE) and homozygous (HO) knock-in mutants using the CRISPR/Cas9 editing system in HCT116 CRC cells. The heterozygous mutant harboring one copy of the SMARCA4-R1157W mutation could more closely mimic the disease state of CRC patients. We then performed the CCK-8, colony formation, and migration assays to investigate the effects of the HE and HO mutations of SMARCA4 on CRC cell growth compared with the wild type. The results showed that the SMARCA4-R1157W HE mutant significantly promoted the CRC cell growth compared with SMARCA4-WT, while the SMARCA4-R1157W HO mutant showed a greater effect on cell proliferation than the SMARCA4-R1157W HE mutant. Consistently, SMARCA4-R1157W HE mutant significantly increased both the transcriptional level and protein level of *EGFR* and *TNS4* compared with SMARCA4-WT, while significantly higher expression levels of *EGFR* and *TNS4* were observed in the SMARCA4-R1157W HO mutant compared with the SMARCA4-R1157W HE mutant (Supplementary Fig. 4j–o). Collectively, these results suggest that the SMARCA4-R1157W mutant reinforces *EGFR* and *TNS4* expression to accelerate CRC cell proliferation.

### The R1157W mutation enhances SMARCA4 ATPase activity and chromatin remodeling ability

**Fig. 4:**
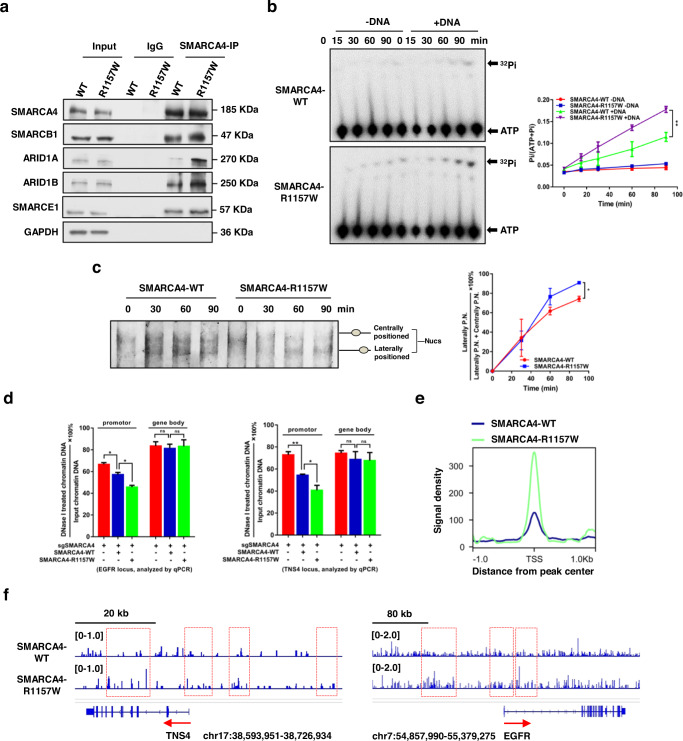
The SMARCA4-R1157W mutation enhances ATPase activity and chromatin remodeling ability. **a** Coimmunoprecipitation assay with lysates prepared from HCT116 cells transfected with SMARCA4-WT or SMARCA4-R1157W plasmids. Anti-SMARCA4 antibody was used for the immunoprecipitation and lysates were immunoblotted for indicated proteins. **b** Time-dependent ATPase activity of SMARCA4-WT and SMARCA4-R1157W were assayed in vitro using γ-^32^P-labeled ATP combined with thin-layer chromatography (left panel). Quantitative analysis of the ATPase activity assay (right panel). **P < 0.01. **c** Nucleosome sliding assay for establishing the remodeling activity of SMARCA4-WT and SMARCA4-R1157W at indicated time points (0, 30, 60, and 90 min) by native gel electrophoresis (left panel) for centrally positioned nucleosomes (P.N.) and laterally positioned nucleosomes. Quantitative analysis of the nucleosome repositioning (right panel). The vertical axis shows the percentage of [the amount of laterally P.N. DNA/(the amount of laterally P.N. DNA + the amount of centrally P.N. DNA)]. *P < 0.05. **d** DNase I chromatin accessibility assay was used to detect the effects of SMARCA4-WT and SMARCA4-R1157W on the accessibility of *EGFR* and *TNS4* genomic regions. Primers were designed on the *EGFR* promoter region (−301 to −132) and exon (4727–4823) to detect the accessibility of *EGFR* loci (left panel). Primers were designed on the *TNS4* promoter region (−400 to −228) and exon (2339–2491) to detect the accessibility of *TNS4* loci (right panel). *P < 0.05, **P < 0.01. **e** Metaplot of ATAC-seq signals around TSSs in SMARCA4-WT or SMARCA4-R1157W HCT116 cells within a ±1 kb window. **f** Genomic tracks of ATAC in the vicinity of the *TNS4* and *EGFR* loci in SMARCA4-R1157W versus SMARCA4-WT HCT116 cells. Track height is normalized to the relative number of mapped reads. In the graphs, the error bars are the standard deviation of the mean.

To probe the underlying mechanism by which SMARCA4-R1157W mutant reinforces EGFR and TNS4 expression, we first determined the effect of the SMARCA4-R1157W mutant on the stability of the SWI/SNF complex by coimmunoprecipitation (Co-IP) experiments. We found that the binding of SMARCA4-R1157W to other key subunits, SMARCB1, ARID1A, ARID1B, and SMARCE1 of cBAF complex, was significantly increased compared to the SMARCA4-WT (Fig. 4a). In contrast, the binding of SMARCA4-R1157W to PBRM1, ARID2, and BRD7 of the PBAF complex or BRD9 of the ncBAF complex was not changed compared to the SMARCA4-WT (Supplementary Fig. 5a). The data suggest that the SMARCA4-R1157W-containing SWI/SNF cBAF complex was more stable than the cBAF complex containing SMARCA4-WT, which was further confirmed by glycerol gradient sedimentation analysis experiments (Supplementary Fig. 5b). Next, we tested whether the SMARCA4-R1157W subunit altered ATPase activity and chromatin remodeling ability of the SWI/SNF complex. Thin-layer chromatography (TLC) assays with γ-^32^P-labeled ATP showed that the ATPase activity of SMARCA4 was significantly augmented in the presence of DNA with the SMARCA4-R1157W mutant compared to the SMARCA4-WT (Fig. 4b). In addition, we used mononucleosomes as substrates to test the chromatin remodeling ability of SMARCA4 by nucleosome sliding assays. In these assays, the position of the nucleosome on the histone octamer would affect the electrophoretic mobility of the DNA fragment, and DNA fragments with laterally positioned and centrally positioned nucleosomes could be separated by native gel electrophoresis. We found that as the incubation time increased, centrally positioned nucleosomes gradually slid laterally, ultimately exposing the nucleosome binding site to other factors for access (Fig. 4c). When the incubation time was increased to 60 or 90 min, the SMARCA4-R1157W mutant further catalyzed lateral sliding of centrally positioned nucleosomes compared to the SMARCA4-WT which suggested an increased remodeling ability (Fig. 4c). To further assess this activity, we examined whether the SMARCA4-R1157W mutant affected chromatin accessibility at the EGFR and TNS4 loci in CRC cells. After the chromatin/DNA samples were pre-exposed to DNase I for digestion, we performed qRT–PCR on the EGFR and TNS4 loci. We found that, relative to SMARCA4 knockout (sgSMARCA4), transfection of SMARCA4-WT or SMARCA4-R1157W rendered the promoters of EGFR and TNS4 more accessible to DNase I digestion (less PCR product) (Fig. 4d). The data indicate that chromatin at the EGFR and TNS4 promoters was accessible while no such accessibility was observed in gene body regions. Notably, compared to SMARCA4-WT cells, SMARCA4-R1157W cells exhibited considerably more accessible chromatin at the EGFR and TNS4 promoters, suggesting that the SMARCA4-R1157W mutant had acquired a stronger chromatin remodeling ability (Fig. 4d). In addition, we performed ATPase assays and determined the *K*_m_, *K*_cat_, and *V*_max_ for both SMARCA4-WT and SMARCA4-R1157W. We found that the SMARCA4-WT mutant displayed over 2-fold higher *V*_max_ and *K*_cat_ values than the SMARCA4-WT protein while similar *K*_m_ values of both proteins were obtained (Supplementary Fig. 5c, d). Furthermore, the SMARCA4-R1157W mutant increased global ATAC accessibility at TSSs, and local ATAC accessibility at promoters, TSSs, and gene body regions of TNS4 and EGFR compared to the SMARCA4-WT (Fig. 4e, f). Taken together, the R1157W mutation in SMARCA4 strengthens the stability of the SWI/SNF complex and enhances SMARCA4 ATPase activity and chromatin remodeling activities in CRC cells.

### SMARCA4-R1157W mutant CRC cells were more sensitive to PRMT1 and SMARCA4 inhibitors which act synergistically to suppress cell proliferation

**Fig. 5:**
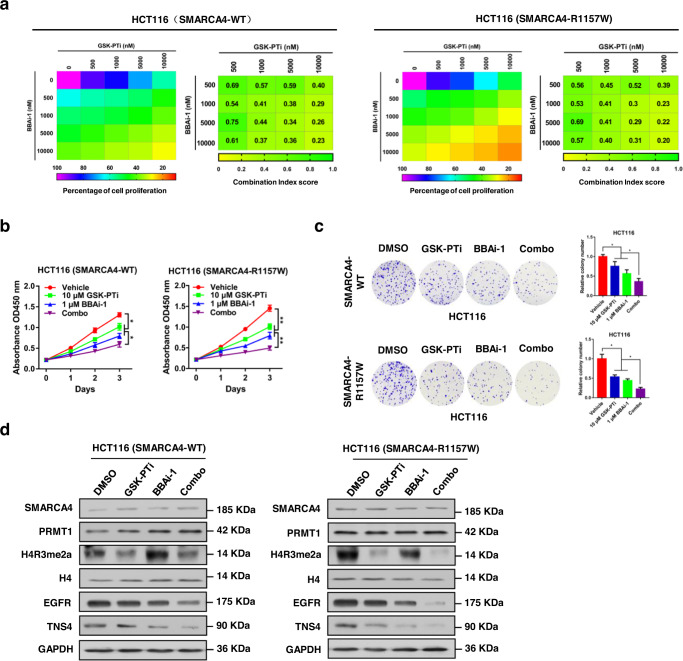
Synergistic effect of combined treatment with GSK-PTi and BBAi-1 in SMARCA4-R1157W HCT116 cells. **a** Dose–response matrix for growth inhibition of SMARCA4-WT (left panels) or SMARCA4-R1157W (right panels) HCT116 cells following 7 days of culture with GSK-PTi plus BBAi-1. Color gradation indicates percent viability at the indicated dose combination. Combination index (CI) scores for SMARCA4-WT (left panels) or SMARCA4-R1157W (right panels) HCT116 cells following 7 days of culture with GSK-PTi plus BBAi-1 at the indicated concentrations. Each CI score represents data from at least three independent experiments. **b**, **c** CCK8 (**b**) and colony formation (**c**) assays were performed to evaluate the effect of the combination treatment with 10 μM GSK-PTi or/and 1 μM BBAi-1 on cell proliferation of SMARCA4-WT or -R1157W HCT116 cells. *P < 0.05, **P < 0.01. **d** Western blot analysis of indicated proteins in SMARCA4-WT or -R1157W mutant HCT116 cells following treatment with 10 μM GSK-PTi or/and 1 μM BBAi-1. Histone H4 and GAPDH were used as loading controls. In the graphs, the error bars are the standard deviation of the mean.

Given that the SMARCA4-R1157W mutant promotes CRC cell proliferation by enhancing the binding to PRMT1-mediated H4R3me2a and chromatin remodeling activities of the SWI/SNF complex, we sought to examine whether the combined inhibition of PRMT1 and SMARCA4 ATPase activity would yield a favorable therapeutic effect on SMARCA4-R1157W mutant CRC. Therefore, we initially examined the effects of a combination of PRMT1 inhibitor, GSK3368715^32^ (referred to as GSK-PTi in this paper) and BRM/BRG1 ATP inhibitor-1^33^ (referred to as BBAi-1 in this paper) on SMARCA4-WT or -R1157W mutant cell proliferation. We found that combined treatment with GSK-PTi with BBAi-1 synergistically enhanced the anti-proliferative effect on SMARCA4-WT or -R1157W mutant HCT116 and SW620 cells, as shown by heatmaps of cell growth inhibition and combination index (CI) score maps (Fig. 5a; Supplementary Fig. 6a). In addition, we used designated concentrations of GSK-PTi (10 μM) and BBAi-1 (1 μM) to confirm the synergistic inhibitory effect of the combination on CRC cell growth. The combination treatment with GSK-PTi and BBAi-1 profoundly hampered the proliferation and colony formation ability of SMARCA4-WT or -R1157W mutant HCT116 (Fig. 5b, c) and SW620 cells (Supplementary Fig. 6b, c). Of note, the combination treatment on SMARCA4-R1157W mutant cells displayed better efficacy than that on SMARCA4-WT cells (Fig. 5b, c; Supplementary Fig. 6b, c). Western blot analyses confirmed that the combination treatment of GSK-PTi and BBAi-1 greatly reduced EGFR and TNS4 expressions in SMARCA4-R1157W mutant cells than those in SMARCA4-WT cells (Fig. 5d; Supplementary Fig. 6d). Although single-drug treatments appeared to work slightly better in SMARCA4-R1157W mutant cells than in SMARCA4-WT cells, there were no statistically significant differences between the two cell lines (Fig. 5b–d). These results indicate that the combination of GSK-PTi and BBAi-1 exerts profound drug synergy in SMARCA4-R1157W mutant CRC cells.

**Fig. 6:**
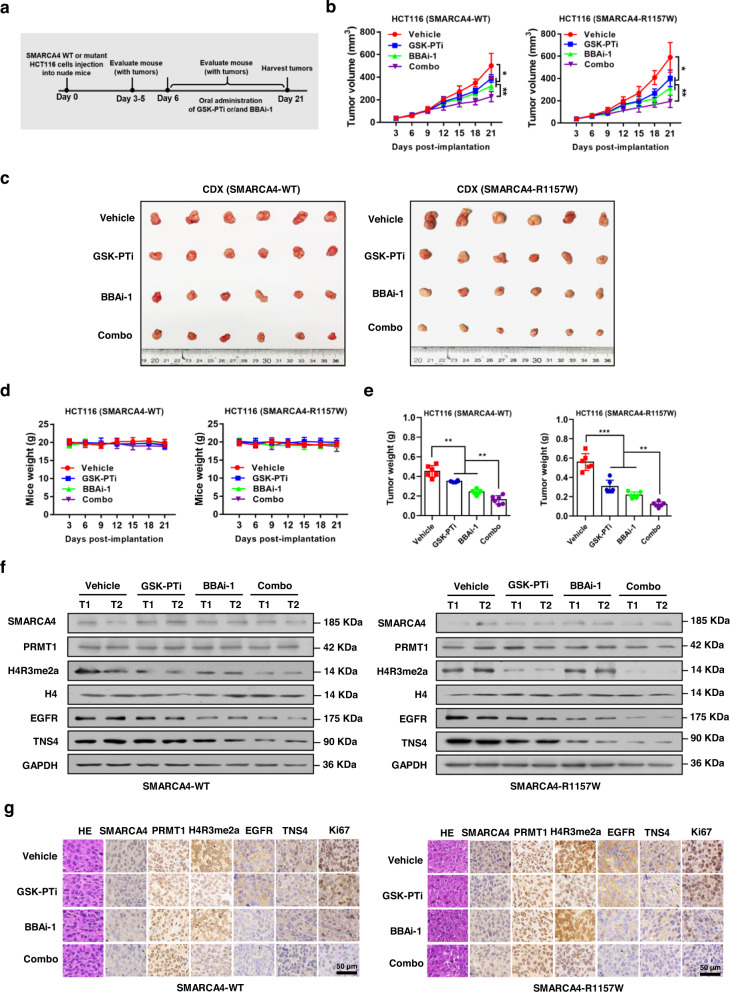
Synergistic effect of combined treatment with GSK-PTi and BBAi-1 in CDX mouse models of CRC. **a** Schematic of the strategy used for testing the efficacy of GSK-PTi and BBAi-1 in vivo. **b**–**e** Tumor volumes (**b**), tumor images (**c**), mouse weights (**d**), and tumor weights (**e**) of SMARCA4-WT or -R1157W HCT116 cell-derived xenograft tumors excised from mice treated with GSK-PTi (25 mg/kg) or BBAi-1 (2.5 mg/kg) or combination. *P < 0.05, **P < 0.01, ***P < 0.001. **f** Western blot analysis of indicated proteins in SMARCA4-WT or -R1157W HCT116 cell-derived xenograft tumors after treatment with GSK-PTi (25 mg/kg) or BBAi-1 (2.5 mg/kg) or combination. **g** Hematoxylin and eosin (H&E) staining and immunohistochemical staining (IHC) analysis with indicated antibodies in SMARCA4-WT or -R1157W HCT116 cell-derived xenograft tumors after treatment with GSK-PTi (25 mg/kg) or BBAi-1 (2.5 mg/kg) or combination. In the graphs, the error bars are the standard deviation of the mean.

To further determine the therapeutic benefits of GSK-PTi and BBAi-1 in vivo, we established SMARCA4-WT or -R1157W mutant HCT116 cell line-derived xenograft (CDX) tumor models. Tumor-bearing mice were treated by oral administration of GSK-PTi (25 mg/kg) and/or BBAi-1 (2.5 mg/kg) for 14 days (Fig. 6a). First, for SMARCA4-WT CDX models, daily single treatment with either GSK-PTi or BBAi-1 had a moderate therapeutic effect, with a tumor growth inhibition (TGI) rate of 23% or 46.9%, respectively, in model mice (Fig. 6b, c, and e). Combination treatment with GSK-PTi and BBAi-1 greatly inhibited tumor growth, showing a TGI rate of approximately 64.6% in the CDX models (Fig. 6b, c and e), but had no effect on mouse body weight (Fig. 6d). Second, for SMARCA4-R1157W mutant CDX models, daily single treatment with GSK-PTi or BBAi-1 had a better therapeutic effect, with a TGI rate of 45.3% or 61.3%, respectively, in model mice (Fig. 6b, c and e). Consistent with the earlier in vitro findings (Fig. 5b and c; Supplementary Fig. 6b, c), combination treatment with GSK-PTi and BBAi-1 greatly inhibited tumor growth, showing a TGI rate of ~78.5% in the CDX models (Fig. 6b, c and e), but had no effect on mouse body weight either (Fig. 6d). As expected, we also detected lower protein levels of EGFR and TNS4 in tumor tissues of SMARCA4-R1157W mutant CDX models after treatment with GSK-PTi and BBAi-1 by western blot (Fig. 6f) and IHC analysis (Fig. 6g) than those in SMARCA4-WT CDX models. Of note, combination treatments in SMARCA4-R1157W mutant model mice showed significantly better effects than those in SMARCA4-WT model mice (Fig. 5b, *P* = 0.03) although there were no statistically significant differences between the two model mouse groups with single-drug treatments (Fig. 5b; *P* = 0.14 or 0.06 for GSK-PTi or BBAi-1 treatment, respectively). Taken together, these data demonstrate that GSK-PTi and BBAi-1 in combination exert profound drug synergy in SMARCA4-R1157W mutant CRC cells.

## Discussion

Cancer genomic studies have shown that subunits of the SWI/SNF chromatin remodeling complex contain a high frequency of genetic alterations among various solid tumors^24^. However, mutations of SMARCA4 and their roles of tumorigenesis in CRC were less characterized. Here, we identified a specific critical mutation R1157W in SMARCA4, the key catalytic subunit in the SWI/SNF complex, with an estimated mutation frequency of 9.4% in human CRC although more patients are required to substantiate this finding. This unexpectedly high mutation frequency may be associated with racial or geographical differences from the Nanjing area in East China. We found that the SMARCA4^R1157W^ mutant increased the binding access to PRMT1-mediated H4R3me2a and enhanced ATPase activity and chromatin remodeling activities, thereby reinforcing *EGFR* and *TNS4* expression and accelerating the progression of CRC. We also showed that the growth of CRC cells and the binding to H4R3me2a were not influenced by SMARCA4-R1157Q or—1243Q mutants. However, further investigations are required to determine whether these mutations might contribute to other aspects of CRC cells (such as differentiation, senescence, apoptosis, metabolism, etc.), and if so, what are possible molecular mechanisms.

Our current study showed that a portion of the SMARCA4 ATPase domain (aa1009–1314) which contains the complete helicase superfamily c-terminal (HELICc) domain (aa1117–1164), endows SMARCA4 with the ability to bind the H4R3me2a modification (Fig. 2e). However, SMARCA2, another core subunit of the SWI/SNF complex with ATPase activity, also harbors a highly conserved HELICc domain (Supplementary Fig. 7a). Interestingly, pulldown assays showed that the HELICc domain of SMARCA2 also bound H4R3me2a in vitro (Supplementary Fig. 7b). It would be interesting to test whether other HELICc domain-containing proteins, such as the CHD family (CHD1, CHD3, CHD4) and INO80^34^ also bind H4R3me2a and have a relevant biological consequence. Previous work has reported mutations (R1105C/H, K1111R/T, A1156T, and G1164R) in the HELICc domain (aa1089–1164) of SMARCA2 in CRC^35,36^, but no missense point mutation corresponding to R1157 of SMARCA4 has been identified. Our identification of a mutation involving R1157 (Supplementary Fig. 7a) suggests that the R1157 residue might be essential to SMARCA4 function, and therefore, is susceptible to interference. Furthermore, the treatment strategy adopted in this study for the inhibition of SMARCA4 ATPase activity by BBAi-1 cannot exclude the possibility that the ATPase activity of SMARCA2 could also be affected. Although there is another SMARCA bromodomain inhibitor, PFI-3, it also could not differentiate between SMARCA2 and SMARCA4^37^. Thus, the development of specific small molecule inhibitors of SMARCA4 is urgently needed. Nevertheless, we found that SMARCA4-R1157W mutant CRC cells were more sensitive to PRMT1 and SMARCA4 inhibitors which act synergistically to suppress CRC progression. The reasons for this could be that the conformational changes around functional sites of the mutated SMARCA4 or/and SWI/SNF-associated complex might alter the accessibility of the inhibitors which warrants further investigation in the future.

The role of SMARCA4 in cancer progression remains controversial, which makes the function of its mutation more complicated. Numerous studies increasingly suggest that SMARCA4 acts as either an oncogene or a tumor suppressor in a cell-type and context-specific manner, although in most cases, SMARCA4 acts as a tumor suppressor^8,13,17,23^. For example, in human lung cancer cell lines, SMARCA4-inactivating mutations are frequently found^38^, and inactivating mutations in SMARCA4 increase the sensitivity to Aurora kinase inhibitors in small cell lung cancer^39^, suggesting that SMARCA4 acts as a tumor suppressor in this context. Similarly, the loss of SMARCA4 ATPase is found specifically for the growth of small cell carcinoma of the ovary-hypercalcemic type (SCCOHT) cell lines^14^, and deleterious mutations in SMARCA4 are identified as the major cause of SCCOHT^40^, also consistent with a role of SMARCA4 as a tumor suppressor. In contrast, our previous work has shown that SMARCA4-WT acts as an oncogene in CRC^23^. In the present study, we showed that the R1157W mutation is an activating oncogenic mutation for CRC progression because the SMARCA4-R1157W mutation significantly increased ATPase activity and chromatin remodeling activities. In line with our results, K1029R and K1033R mutations in SMARCA4 were also identified as gain-of-function mutations from in vitro biochemical studies^41^. More importantly, we have shown that the SMARCA4-R1157W mutant enhanced SMARCA4 binding to PRMT1-mediated H4R3me2a, and increased its enrichment at the EGFR and TNS4 promoters, reinforcing their transcriptional expression and accelerating CRC progression. These results are consistent with our previous findings that EGFR and TNS4 are major target genes of SMARCA4^23^. Thus, despite its diverse roles, our studies support SMARCA4 acting as an oncogene for CRC.

Since the first targeted agent for CRC, the anti-EGFR agent cetuximab, was approved by the FDA in 2004, the antiangiogenic agent bevacizumab and the immune checkpoint inhibitor nivolumab have been launched successively^42^. Targeted therapy has successfully prolonged the survival of CRC patients, and numerous agents that block different critical pathways are being developed and commercialized at a rapid pace. In the current study, we found that dual inhibition of PRMT1 and SMARCA4 resulted in profound synergistic anti-neoplastic activity against SMARCA4-R1157W mutant CRC cells in vivo and in vitro. Intriguingly, CRC tumors with high expression levels of SMARCA4-WT could also benefit from combination therapy. These findings may provide a rationale for developing a new personalized therapeutic strategy for CRC patients with tumors carrying the SMARCA4-R1157W mutation.

In summary, our study reveals that the SMARCA4-R1157W mutation accelerates the progression of CRC by increasing the SMARCA4 binding to PRMT1-mediated H4R3me2a and enhancing ATPase activity and chromatin remodeling activities. The synergistic effect of inhibiting both PRMT1 and SMARCA4 (as with GSK-PTi and BBAi-1) in SMARCA4-R1157W mutant CRC cells may shed new light on the possibility of precision therapy for CRC patients with SMARCA4-R1157W mutation.

## Methods

### Cell lines and cell culture

Colorectal cancer cell lines (HCT116 and SW620) were obtained from the Shanghai Institute of Cell Biology, Chinese Academy of Sciences (Shanghai, China). The cells were maintained at 37 °C in a 5% CO_2_ incubator in McCOY’s 5A medium (HCT116) or RPMI-1640 (SW620) supplemented with 10% FBS (Life Technologies). The cell lines were authenticated by Genetic Testing Biotechnology Corporation (Suzhou, China) using short tandem repeat (STR) profiling and routinely tested to exclude mycoplasma contamination.

### Generation of SMARCA4 knockout and SMARCA4-R1157W mutant knock-in cells

Lentiviral CRISPR/Cas9‐mediated SMARCA4 knockout vectors were constructed by cloning SMARCA4 guide RNA:

sgRNA1 F: 5’‐CACCGTCGCGCTCATCACGTACCTC‐3’;

R: 5’‐AAACGAGGTACGTGCTGAGCGCGAC‐3’;

sgRNA2 F: 5’‐CACCGTCACAGTGTCTGCCGACTGG‐3’;

R: 5’‐AAACCCAGTCGGCAGACACTGTGAC‐3’;

into the BsmBI site of the lentiviral vector LentiCRISPR V2. Lentivirus was produced by packaging in 293T cells. Stable pools of SMARCA4‐KO cells were generated by transducing HCT116 or SW620 cells with lentiviral CRISPR/Cas9 vectors and selected with 4 μg/ml puromycin. Surviving cells were cloned by limiting dilution and then used in experiments after validation by immunoblotting.

The CRISPR/Cas9 editing system was used to generate the SMARCA4-R1157W mutant knock-in HCT116 cells. The donor sequence is

TCTGAGTACTTCATCTTCCTGCTCAGCACCTGGGCTGGGGGGCTCGGCCTGAATCTCCAGTCGGCAGACACTGTGATCATTTTT.

Cells transfected with the sgRNA2 plasmid and the donor sequence were screened with 4 μg/ml puromycin. Surviving cells were cloned by limiting dilution and SMARCA4-R1157W heterozygous and homozygous mutant cells were obtained by DNA sequencing.

### Culture of CRC patient-derived organoids

Fresh CRC tissue samples were processed as previously described^43^, with several modifications. The tissue was cut into ~5 mm pieces, washed with ice-cold PBS at least ten times, and subsequently digested with Gentle Cell Dissociation Reagent (#07174, STEMCELL Technologies) for 60 min at 37 °C on a rocking platform set at medium speed (~40 rpm). The cells were centrifuged at 290×*g* for 5 min, and the supernatant was collected. The cell pellet was suspended in Matrigel (#356231, growth factor reduced, Corning) and dispensed into 24-well culture plates (50 μl Matrigel/well). Then, 800 μl of Human Organoid Growth Medium (#06010, STEMCELL Technologies) was added to each well and incubated at 37 °C in a 5% CO_2_ incubator. Media were changed every 2 or 3 days, and cells were passaged after 7–14 days.

### Plasmid construction, recombinant protein expression, and purification

pGEX-6p-1 plasmids encoding PRMT1, wild-type or mutant (R1157W, R1157Q, R1243Q) SMARCA4-F4 (aa1009–1314) were transformed into *E. coli* BL21 and induced with IPTG at 25 °C for 16 h. After induction, the bacterial cells were collected by centrifugation, suspended in cold PBS, and lysed by sonication. The GST-fusion proteins were affinity-purified with glutathione-Sepharose beads (GE Healthcare) according to the user’s manual and assessed by SDS–PAGE. The Flag-SMARCA4 full-length coding regions (wild-type or R1157W mutant) were cloned into the eukaryotic expression plasmid pCMV5. The SMARCA4 KO wild-type or mutant rescue plasmids were synonymously mutated in CRISPR/Cas9 sgRNA2 target sequences (CAAAGTGCCGATACAGTA instead of CAGTCGGCAGACACTGTG) and inserted into the lentiviral vector pLVX-IRES-mCherry. All plasmids constructed above were confirmed by DNA sequencing.

### Microscale thermophoresis (MST), isothermal titration calorimetry (ITC) assays, and peptide pulldown assays

MST measurements were performed on a NanoTemper Monolith NT.115 (NanoTemper Technologies GMBH) at 60% LED and an MST power of 20%. The purified recombinant SMARCA4-F4 (aa1009–1314) wild-type and R1157W mutant proteins were labeled with NT-647 dyes. The concentration of the labeled proteins was 100 nM in PBS, 0.05% Tween-20, while the concentration of H4R3me2a peptide ranged from 10 nM to 500 μM. Measurements were performed at 25 °C. Dissociation constants were calculated by NanoTemper Analysis 1.5.41 software using the mass action equation (*K*_d_ formula).

ITC measurements were carried out at 25 °C using the MicroCal ITC-200 system (Malvern Instruments Ltd.). The H4R3me2a peptide and proteins were all subjected to extensive dialysis against ITC buffer (20 mM Na_3_PO_4_, pH 6.8, 200 mM NaCl, 1 mM TCEP). Peptide at a concentration of 1 mM was placed in the syringe, and proteins at a concentration of 50 μM were loaded into the ITC cell. Following the preinjection, the subsequent 19 injections of the sample (2 μl each) were analyzed. The data were further processed using the numerical model implemented in the Origin 7.0 software package (Origin Lab).

Peptide pulldown assays were performed as described previously^44^. COOH-terminal biotin-tagged 20-mer N-terminal peptides of histone H4 with the Arg3 residue unmethylated, symmetrically methylated, or asymmetrically methylated were used in the assay.

### RNA isolation and qRT–PCR analysis

Total RNA was extracted from cells using TRIzol reagent (Invitrogen). Synthesis of complementary DNAs (cDNAs) was completed using a HiScript 1st Strand cDNA Synthesis Kit (Vazyme Biotech, China). Quantitative RT–PCR was performed using AceQ qPCR SYBR Green Master Mix (Vazyme Biotech) according to the manufacturer’s protocols. Experiments were performed using a StepOnePlus^TM^ Real-Time PCR System (Thermo Scientific). Relative mRNA levels of target genes were normalized to the expression of the reference gene GAPDH, and each reaction was performed in triplicate. The primer sequences for RT–PCR is listed in Supplementary Table 2.

### Western blot analysis

Cellular proteins were extracted using cell lysis buffer for Western blots (P0013, Beyotime). All proteins were separated by SDS–PAGE, followed by semidry electroblotting onto PVDF membranes (Roche). Primary antibodies against SMARCA4 (ab110641, 1:1000, Abcam), Flag tag (AE005, 1:1000, Abclonal), EGFR (ab52894, 1:1000, Abcam), TNS4 (ab99887, 1:1000, Abcam), GST tag (AE001, 1:1000, Abclonal), SNF5 (#191735, 1:1000, Cell Signaling Technology), ARID1A (#12354, 1:1000, Cell Signaling Technology), ARID1B (#92964, 1:1000, Cell Signaling Technology), BAF57 (#33360, 1:1000, Cell Signaling Technology), and GAPDH (A19056, 1:1000, Abclonal), PBRM1 (ab305223, 1:1000, Abcam), ARID2 (ab245529, 1:1000, Abcam), BRD7 (ab270982, 1:1000, Abcam) and BRD9 (ab259839, 1:1000, Abcam) were used. HRP-conjugated goat anti-rabbit IgG (AS014, 1:10,000, Abclonal) and goat anti-mouse IgG (AS003, 1:10,000, Abclonal) were used as the secondary antibodies. Blots were exposed to X-ray film after development using High-sig Western ECL Blotting Substrate (Tanon, Shanghai), and the films were scanned as image files using an Epson Perfection V700 Photo Scanner.). All western blots within a panel are from the same experiment and all blots were processed in parallel. For uncropped images and scans of the blots, refer to Supplementary Figs. 8–18.

### Glycerol gradient sedimentation analysis

SMARCA4-associated proteins were purified by performing Flag-IP in HCT116 cells using anti-Flag M2 affinity gel and eluted by competition using 3×Flag peptide (Beyotime Biotechnology, Beijing, China). One milliliter of glycerol (final concentration 15–35%-20 mM Tris pH 7.5, 0.15 M KCl, 2.5 mM MgCl_2_, 0.1% Tween, 0.05% NP-40) was layered into centrifugation tubes (13 × 51 mm Ultra-Clear Tubes, Beckman), and then a linear gradient was obtained after 12 h of diffusion at 4 °C. The eluted protein complexes were loaded on top of the glycerol gradient, and fractionated by ultracentrifugation at 40,000 rpm for 12 h at 4 °C. Fractions of 200 μL were collected from the top of the gradient and an equal volume of fractions was analyzed by western blotting with specific antibodies.

### Chromatin immunoprecipitation (ChIP) assay

ChIP assays were performed with HCT116 cells as described previously^45^. Chromatin fractions from HCT116 cells were immunoprecipitated with specific antibodies as indicated in the text. Normal rabbit IgG (A7016, Beyotime) served as a control. The final ChIP DNAs were analyzed by quantitative real-time PCR with primers that encompassed the promoter region of genes of interest. The primer sequences are listed in Supplementary Table 3.

### CCK8, colony formation, and migration assays

Cell proliferation was assessed using the CCK-8 (Vazyme Biotech) assay. Cells were seeded into 96-well plates at a concentration of 1000 cells/well. At different time points (0, 24, 48, and 72 h) following incubation at 37 °C, the CCK-8 assay was carried out by adding 10 µl CCK-8 reagent to each well. Cell proliferation was determined by measuring the absorbance at 450 nm using a microplate reader (Safire, TECAN). For the colony formation assay, cells were seeded in triplicate at a density of 1000 cells per well in six-well plates. After culturing for ~2 weeks, the colonies were fixed with methanol and stained with 0.1% crystal violet (Sangon Biotech, China). Migration assays were performed with a pore size of 8 µm (Corning, USA) in the Transwell chamber. A total of 1 × 10^5^ cells were seeded into the upper chamber, and medium supplemented with 20% FBS was added to the bottom chamber. After incubation for 24 h, the cells remaining on the upper surface that did not pass through the membranes were removed carefully with a cotton swab. Cells that migrated through the membranes were fixed in 100% methanol for 10 min, stained with 0.1% crystal violet for 15 min, and washed with PBS. Values for migration were evaluated by counting five fields per membrane under a microscope (Nikon) at ×100 magnification.

### Protein structure homology modeling

To evaluate the effect of SMARCA4-R1157W mutant protein on the affinity of the H4R3me2a peptide, three-dimensional (3D) structural homology modeling of the human SMARCA4-F4 fragment (aa1009–1314) was generated by submitting it to the SWISS-MODEL automated structure homology-modeling server. The SMARCA4 protein homology model was built based on the structures of MtISWI (PDB 5JXR) and the Arg3 residue was asymmetrically demethylated (20 amino acid residues in the N-terminus) based on the structure of the H4 peptide (PDB 2KWO). The optimized SMARCA4-WT and -R1157W mutant proteins were docked with the H4R3me2a peptide to analyze the changes in binding free energy, intermolecular interaction force, protein structure, and charge distribution. The figures were generated using PyMOL (The PyMol Molecular Graphics System, Version 2.0 Schrödinger, LLC).

### CUT & Tag analysis and ATAC-seq analysis

CUT & Tag assays were performed using Vazyme CUT & Tag assay Kit (Vazyme Biotech, Nanjing, China). ~10,000 intact cell nuclei from each sample were used for CUT & Tag assays according to the Vazyme user manual. Sequencing data were analyzed and visualized with Integrative Genomics Viewer software.

Fifty thousand cells were collected and centrifuged. The unfixed nuclei were tagged using tn5 transposase and the resulting library fragments were generated by 12 PCR cycles. Sequencing was performed on an Illumina HiSeqXten-PE150 by LC-Bio Technology (Hangzhou, China). Visualization of peak distribution along genomic regions of genes of interest was performed with Integrative Genomics Viewer software.

### ATPase assays and nucleosome sliding assays

The ATPase assays were performed as described previously with modifications^46^. The 20 μl reactions contained 50 mM Tris (pH 7.5), 50 mM NaCl, 8 mM MgCl_2_, 0.5 mM PMSF, 2 mM ATP, 2 mM DTT, and 1 μg of a 1 kb DNA fragment. The reaction was initiated by adding 1 μl of 10 mCi/ml [γ-^32^P] ATP (3000 Ci/mmole; PerkinElmer) and 1 μg of SMARCA4 wild-type or mutant proteins. The reactions were then incubated at 30 °C for the indicated times (0, 15, 30, 60, and 90 min) and were stopped by the addition of 5 μl of 500 mM EDTA. One microliter of the reaction mixture was spotted onto PEI-cellulose TLC plates (Merck), and chromatography was run in 0.5 M LiCl, 1 M formic acid. The ratio of inorganic phosphate to ATP at each time point was quantified using a Molecular Dynamics Phosphorimager (Amersham Biosciences) and ImageQuant TL software.

The nucleosome sliding assay was performed as previously described with several modifications^41,47^. A 255 bp DNA template from the Widom 601 positioning sequence^48^ was assembled into mononucleosomes by the “salt jump” method in the presence of core histones isolated from HeLa cells^49^. Mononucleomes (100 ng DNA) were incubated with wild-type or mutant SMARCA4 proteins (100 ng) in 40 μl reactions containing 20 mM HEPES–NaOH (pH 7.9), 50 mM MgCl_2_, 1 mM DTT, 0.1 mM PMSF, 0.1 mg/ml BSA, 3% glycerol, 0.02% NP-40, 0.02% Triton X-100, 2 mM ATP at 30 °C for 0, 30, 60, or 90 min. Reactions were quenched by the addition of 20 mM EDTA and 1% glycerol. Samples were resolved by 6% native polyacrylamide/0.5×TBE gel electrophoresis and visualized by SYBR green staining (Invitrogen).

An ATPase activity assay kit (#MAK113, Sigma,) was used to measure the Kinetic parameters (*V*_max_, *K*_m_, and *K*_cat_) of SMARCA4 on ATP. In detail, SMARCA4-WT and -R1157W mutant proteins were incubated with saturating amounts of plasmid DNA and various concentrations of ATP. Reaction rates were determined by quantitating the fraction of ATP hydrolyzed at multiple time points. Kinetic parameters were determined by fitting the initial rate data to the Michaelis–Menten equation using the Origin software. All reactions were performed in triplicate.

### DNase I chromatin accessibility analysis

The DNase I chromatin accessibility assay was performed as previously described^50,51^. The isolated chromatin was digested with DNase I (NEB) at 3 U/100 µl for 8 min at room temperature. DNA was lightly sonicated (10 cycles of 30 s on/20 s off) using a Bioruptor Plus (Diagenode). The purified DNA was used for qRT–PCR and the results were analyzed according to the formula 100/2^Ct (DNase I)−Ct (no DNase I)^. The primer sequences for qRT–PCR is listed in Supplementary Table 4.

### In vivo drug studies

To establish cell line-derived xenograft (CDX) mouse models, 6-week-old BALB/c female nude mice were obtained from the Model Animal Research Center of Nanjing University (Nanjing, China). All animal experimental procedures were approved by the Animal Ethical and Welfare Committee of Nanjing University. Approximately 5 × 10^6^ SMARCA4 wild-type or R1157W mutant HCT116 cells were injected subcutaneously into the right flank of each mouse. The mice were randomized into 4 groups (6 mice/group) when the tumor volume reached approximately 80 mm^3^ (volume = 0.52 × length × width^2^), at which point drug dosing every day was initiated. GSK-PTi (GSK3368715, HY-128717A, MCE)^32^ and BBAi-1 (BRM/BRG1 ATP inhibitor-1, HY-119374, MCE)^33^ were dissolved in 10% DMSO, 90% saline (containing 20% SBE-β-CD). GSK-PTi (25 mg/kg) and BBAi-1 (2.5 mg/kg) were administered orally. Body weight and tumor volume of the mice were measured every two days. Treatments were continued for 14 days, after which all mice were euthanized for analysis. Tumor tissues were weighed and prepared for immunoblotting or immunohistochemical staining. Tumor growth inhibition (TGI) rate was measured using the formula: TGI (%) = [1−(*V*_t_−*V*_0_ in treated group)/*V*_t_−*V*_0_ in vehicle group)]×100 where *V*_t_ is the volume on each day and *V*_0_ is the volume at the beginning of the treatment.

### Clinical samples and immunohistochemistry (IHC)

Fresh, matched CRC tissues and adjacent normal tissues used for biological analyses and organoid establishment were obtained from Jiangsu Province Hospital of Chinese Medicine (Nanjing, China). All patients gave written informed consent for participation in this study. The study on fresh clinical samples was approved by the ethics committee of Jiangsu Province Hospital of Chinese Medicine.

IHC was performed by Nanjing Microworld Biotechnology Co., Ltd. using paraffin-embedded, formalin-fixed tumor tissues removed from the tumor-bearing nude mice at the end of the study. Sections were incubated with antibodies specific for PRMT1 (Proteintech, 11279–1, 1:100), H4R3me2a (PTMBio, PTM667, 1:100), EGFR (Abcam, ab52894, 1:200), TNS4 (Abcam, ab99887, 1:200) and SMARCA4 (Abcam, ab110641, 1:100). Immunocomplexes were visualized using a 2-Solution DAB Kit (Invitrogen).

### Combination inhibitor treatment for synergy in vitro

HCT116 and SW620 cells were treated with GSK-PTi, BBAi-1, or a combination of GSK-PTi and BBAi-1 for 6 days in 96-well plates, and cell viability was measured by CCK-8 assay. To determine the presence of a possible synergistic effect of GSK-PTi and BBAi-1, the combination index (CI) was calculated by CompuSyn Version 1.0 software. CI < 0.85 indicates synergy, and CI > 1.2 indicates antagonism.

### Statistical analysis

All experiments were performed in triplicate, and the results are presented as the mean ± standard deviation unless otherwise indicated. Statistical analyses were conducted using GraphPad Prism 7.0 software (GraphPad Software, San Diego, CA, USA). Significant differences between the two groups were evaluated using two-tailed independent Student’s *t*-tests or Fisher’s exact test. *P* < 0.05 was considered significant.

## Supplementary information

Supplemental data

## Data Availability

The datasets used and/or analyzed in the current study are available from the corresponding author on reasonable request. CUT & Tag data have been deposited in the GEO database with the accession number GSE212334 and the ATAC-seq data have been deposited in the GEO database with the accession number GSE221947.
