# Supplementary material for: The SMARCA4R1157W mutation facilitates chromatin remodeling and confers PRMT1/SMARCA4 inhibitors sensitivity in colorectal cancer
Source: NPJ Precis Oncol. 2023 Mar 15;7:28. doi: 10.1038/s41698-023-00367-y (PMC10017700; doi:10.1038/s41698-023-00367-y)
Supplement: Supplementary file 1 — Supplemental data [file 41698_2023_367_MOESM1_ESM.pdf]

Supplementary Information

Supplementary Table 1. Clinicopathologic characteristics of SMARCA4-WT (wild-type) and SMARCA4-R1157W mutant in CRC patients.

Characteristics	SMARCA4		*P* value
	WT	R1157W	
Gender			
Male	41	5	0.667
Female	17	1	
Age			
＞60	39	3	0.4055
⩽60	19	3	
Grade			
T1-T2	50	2	0.0093
T3	8	4	
TNM stage			
I-II	33	2	0.3975
III-IV	25	4	

Analysis of pathological parameters in patients with CRC. Six samples with SMARCA4-R1157W mutation were identified in 64 CRC samples by direct sequencing. According to the TNM staging criteria set by the American Joint Committee on Cancer (AJCC), patient information was classified by gender, age, grade, and stage. *P* values were derived using Fisher's exact test to compare values for the two parameters in each category. [Grade T1-T2: the cancer has grown into submucosa (T1) or muscularis propria (T2). Grade T3: the cancer has spread into subserosa. Stage I-II: the cancer has spread to surrounding tissues, but not to lymph nodes. Stage III-IV: the cancer has spread to lymph nodes and is more likely to spread to distant sites such as liver, lung, peritoneum, or ovaries].

Supplementary Table 2. Primers for RT-PCR

Gene names	Sequence (5’-3’)
SMARCA4-F	GACCAGCACTCCCAAGGTTAC
SMARCA4-R	CTGGCCCGGAAGACATCTG
EGFR-F	AGGCACGAGTAACAAGCTCAC
EGFR-R	ATGAGGACATAACCAGCCACC
TNS4-F	AGGACACCAGAACTCCGTTCA
TNS4-R	TCTCGGGTGATGTTTGGCTTA
GAPDH-F	GAAGGTGAAGGTCGGAG
GAPDH-R	GAAGATGGTGATGGGATTTC

Supplementary Table 3. Primers for ChIP

Gene names	Sequence (5’-3’)
EGFR-ChIP-F	CTCCTCAGGGCACCCGCTC
EGFR-ChIP-R	CCAGGCGGCGGAGGAGGGATC
TNS4-ChIP-F	CACTGGCATCCTGCTTTGTCAAG
TNS4-ChIP-R	GTTCCTGCTTGGGCTTCTCGTG

Supplementary Table 4. Primers for DNase I chromatin accessibility analysis

Gene names	Sequence (5’-3’)
EGFR-F (promoter)	CTCCTCAGGGCACCCGCTC
EGFR-R (promoter)	CCAGGCGGCGGAGGAGGGATC
EGFR-F (gene body)	GCCTTGACTGAGGACAGCAT
EGFR-R (gene body)	CCTTACGCCCTTCACTGTGT
TNS4-F (promoter)	CACTGGCATCCTGCTTTGTCAAG
TNS4 -R (promoter)	GTTCCTGCTTGGGCTTCTCGTG
TNS4 -F (gene body)	GAGAGACACGAAGCAGCAGT
TNS4-R (gene body)	CCAGTCTTGGAGAAGCCACC

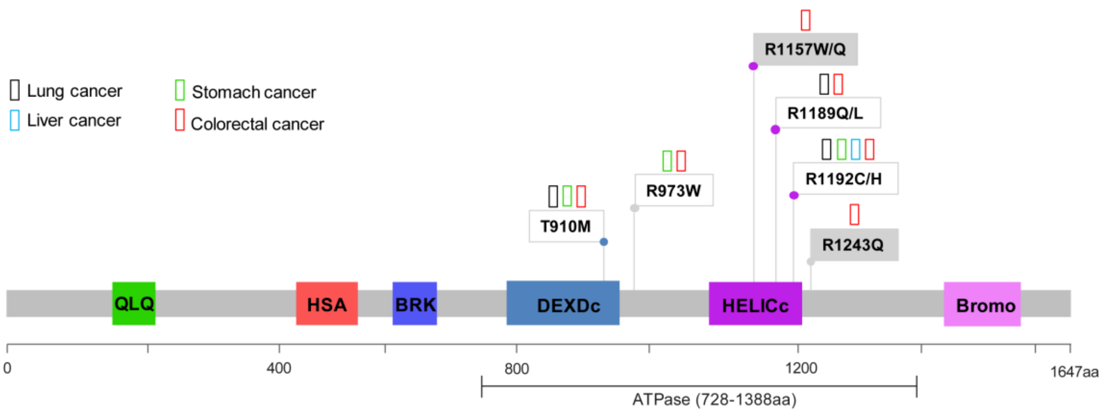

Supplementary Figure 1. SMARCA4 ATPase domain harbors specific genomic mutations in CRC. Mutations of SMARCA4 in lung, liver, stomach, and colorectal cancers were analyzed in the cBioPortal database.

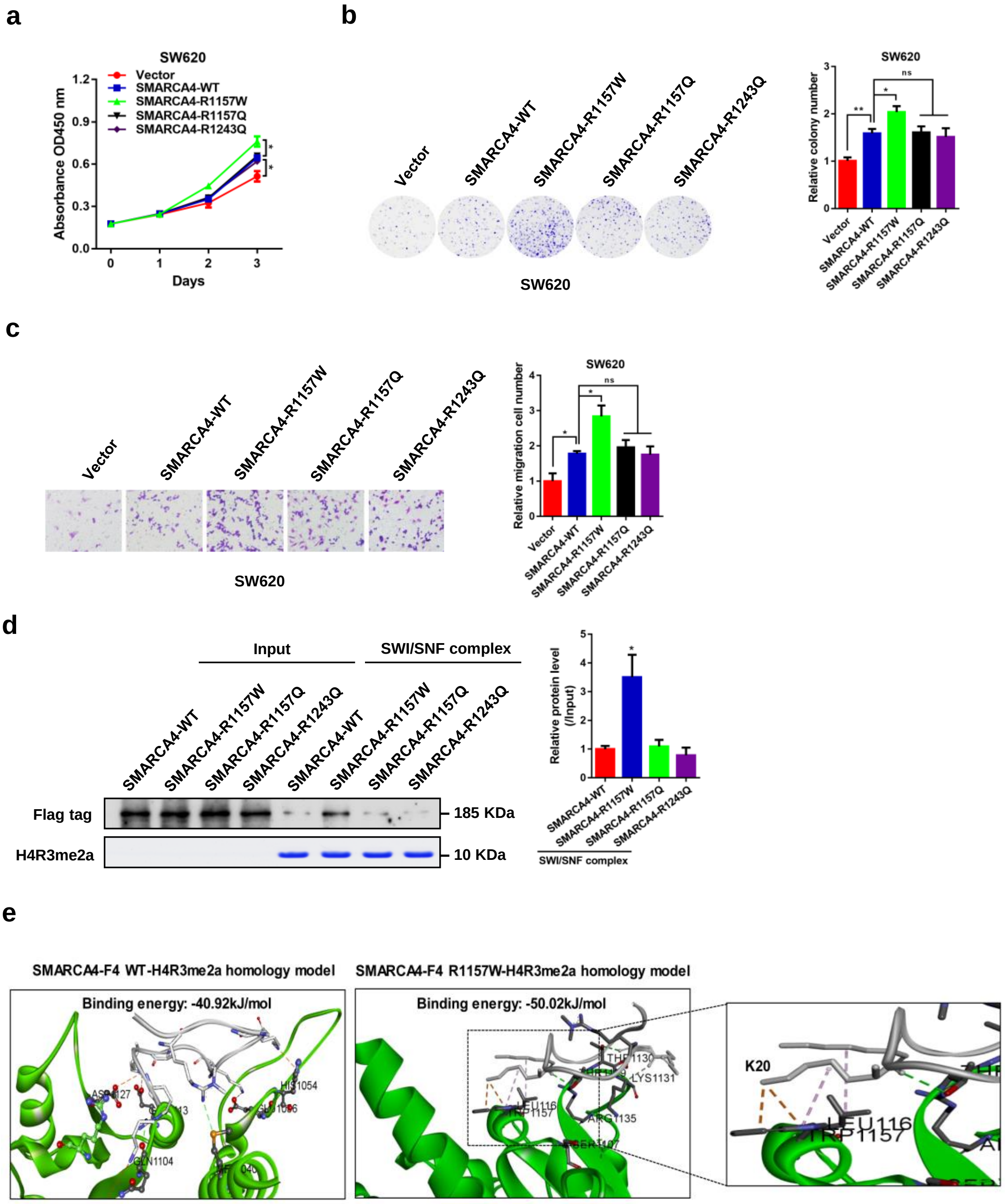

Supplementary Figure 2. The SMARCA4-R1157W mutant promotes the growth of SW620 cells. a Proliferation of SW620 cells transfected with SMARCA4 overexpression plasmids (SMARCA4-WT, SMARCA4-R1157W, SMARCA4-R1157Q, or SMARCA4-R1243Q) or vector plasmids. Values at the indicated time points represent mean ± SD from three independent tests. *$P < 0.05$. **b** Colony formation assay of SW620 cells transfected with SMARCA4 overexpression

plasmids as in (A). Representative images (left panel) and quantitative analyses of the colony formation (right panel) are shown. *$P < 0.05$, **$P < 0.01$. **c** Migration assays of SW620 cells transfected with SMARCA4 overexpression plasmids as in (A). The numbers of migrated cells were quantified by counting the numbers of cells in entire fields at ×200 magnification. Representative images (left panel) and quantitative analyses of the migrated cells (right panel) are shown. *$P < 0.05$. **d** pLVX-IRES-mCherry-Flag-SMARCA4-WT and -R1157W/R1157Q/R1243Q mutant plasmids were stably transfected into SMARCA4-KO HCT116 cells. The Flag-SMARCA4 protein and its co-assembled SWI/SNF complex were immunoprecipitated by anti-Flag M2 affinity gel, and then eluted by competition using 3 x Flag peptide. The purified complexes were then subjected to a pulldown assay with biotin-labeled H4R3me2a peptide (left, top panel). Coomassie blue staining shows equivalent loading of the H4R3me2a peptide (left, bottom panel). Quantitative analysis of the peptide pull-down assay (right panel). *$P < 0.05$. **e** The docking of SMARCA4-WT and -R1157W mutant proteins with the H4R3me2a peptide was mimicked by protein structure homology modeling, and the binding energy, intermolecular interaction force, protein structure and charge distribution were analyzed. In the graphs, the error bars are the standard deviation of the mean.

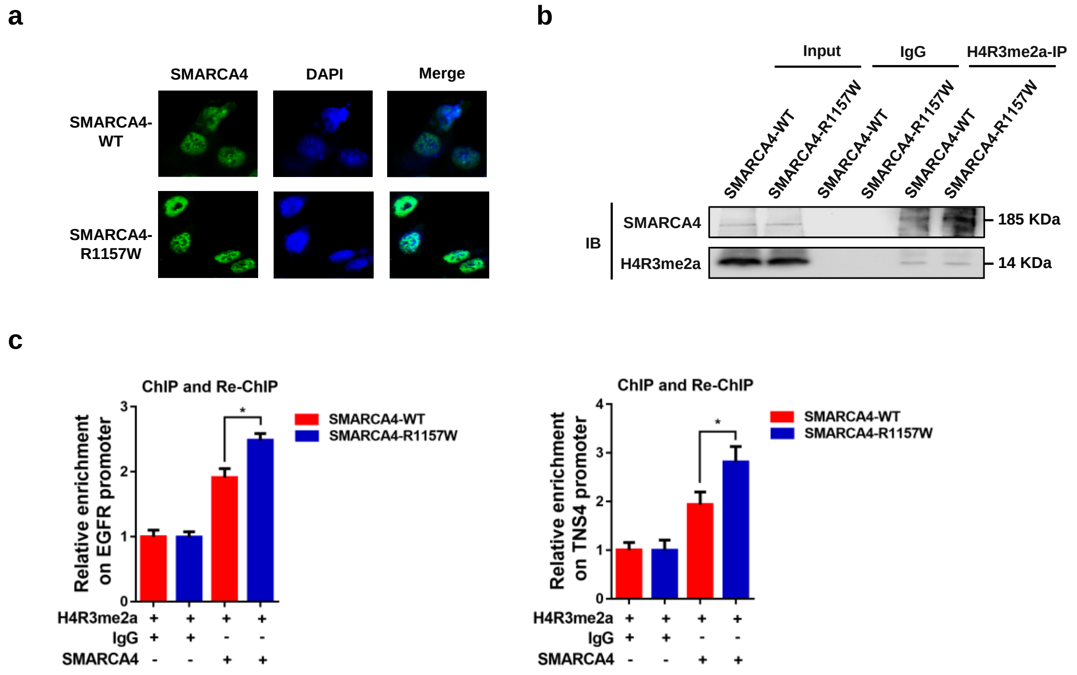

Supplementary Figure 3. The SMARCA4-R1157W mutation enhances its binding to H4R3me2a. a Immunofluorescence assay was performed in HCT116 cells after transfection with SMARCA4-WT or SMARCA4-R1157W plasmids; rabbit anti-SMARCA4 antibody (green), DAPI (blue). **b** Coimmunoprecipitation assay with lysates prepared from SMARCA4-KO HCT116 cells after transfection with SMARCA4-WT or -R1157W mutant plasmids. Anti-H4R3me2a antibody was used for immunoprecipitation, and precipitates were blotted with the indicated antibody. IgG serves as a negative control. **c** ChIP-reChIP analysis of chromatin from SMARCA4-KO HCT116 cells after transfection with SMARCA4-WT or R1157W mutant plasmids. The first antibody (H4R3me2a) and second antibody (SMARCA4) used in the reChIP are shown below the bar plot. The amount recovered from the reChIP was determined by qPCR and is shown as values relative to IgG. *$P < 0.05$. In the graphs, the error bars are the standard deviation of the mean.

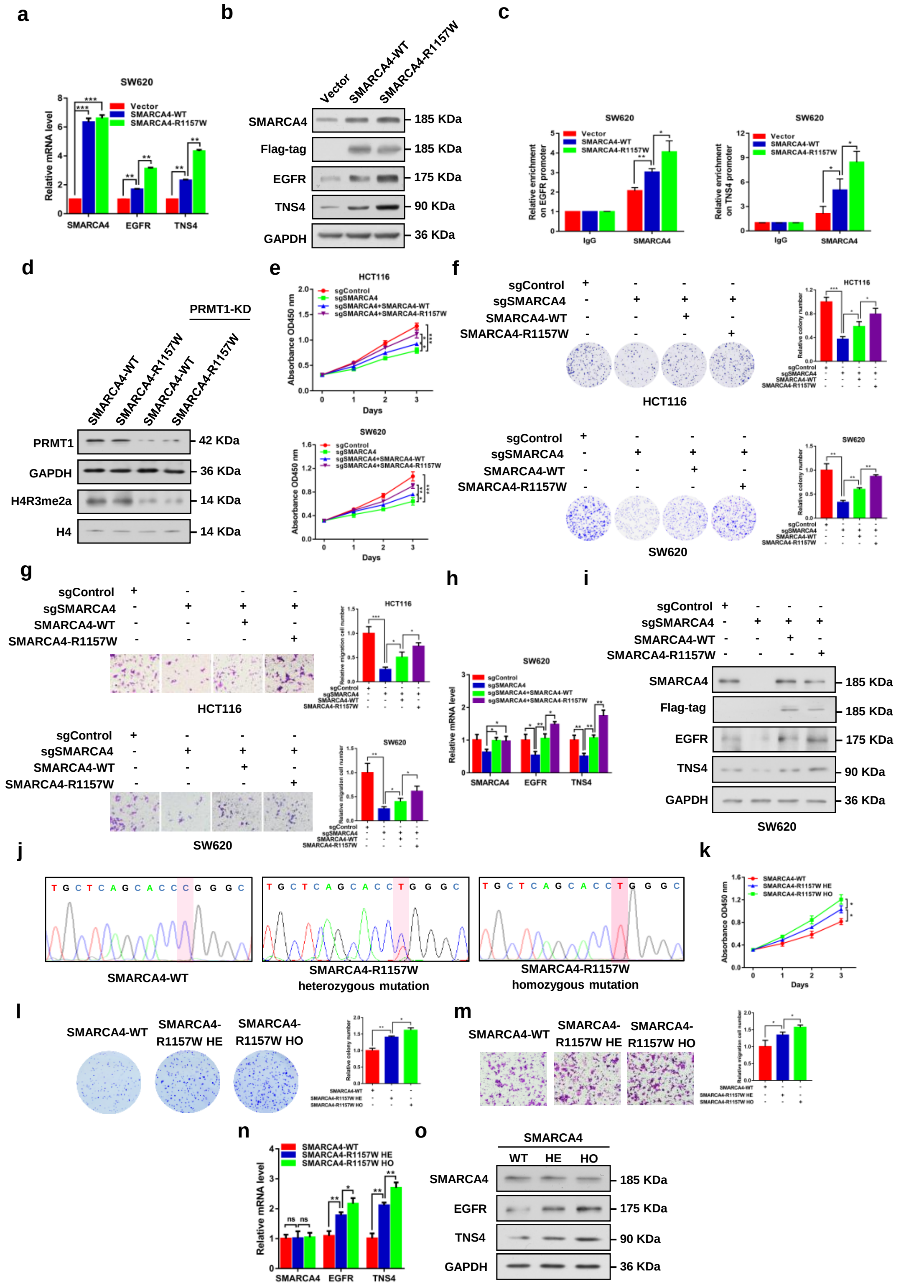

Supplementary Figure 4. The SMARCA4-R1157W mutant reinforces expression of *EGFR* and *TNS4* in SW620 cells. a Quantitative real-time PCR analysis of *SMARCA4*, *EGFR*, and *TNS4* mRNA levels normalized to GAPDH in SW620 cells

transfected with SMARCA4-WT or R1157W overexpression plasmids. **$P < 0.01$, ***$P < 0.001$. **b** Western blot analysis of indicated proteins in SW620 cells transfected with SMARCA4-WT or SMARCA4-R1157W overexpression plasmids. GAPDH served as a loading control. **c** ChIP analysis of SMARCA4-WT and SMARCA4-R1157W binding to the EGFR and TNS4 promoter in SW620 cells. *$P < 0.05$, **$P < 0.01$. **d** Western blot analysis of the indicated proteins in SMARCA4-WT or SMARCA4-R1157W HCT116 cells, and SMARCA4-WT or SMARCA4-R1157W HCT116 cells with PRMT1-knockdown. GAPDH and histone H4 served as a loading controls. **e-g** CRISPR/Cas9 was used to knockout SMARCA4 in HCT116 or SW620 cells, and SMARCA4-WT or SMARCA4-R1157W plasmids were transfected into SMARCA4-KO cells. CCK-8 assay (**e**) was used to detect proliferation of cells in each group. Colony formation assay (**f**) was used to detect the clonogenesis of cells in each group. Migration assay (**g**) was used to detect migration of cells in each group. *$P < 0.05$, **$P < 0.01$, ***$P < 0.001$. **h** Quantitative real-time PCR analysis of *SMARCA4*, *EGFR*, and *TNS4* mRNA levels normalized to *GAPDH* in SMARCA4-KO SW620 cells transfected with SMARCA4-WT or SMARCA4-R1157W plasmids. *$P < 0.05$, **$P < 0.01$, ***$P < 0.001$. **i** Western blot analysis of indicated proteins in SMARCA4-KO SW620 cells transfected with SMARCA4-WT or SMARCA4-R1157W plasmids. GAPDH served as a loading control. **j** DNA sequencing confirmed the knock-in of SMARCA4-R1157W heterozygous mutation (HE) and homozygous mutation (HO). **(k-m),** CCK-8 assay (**k**) was used to detect the proliferation of SMARCA4-WT, -R1157W HE and -R1157

HO HCT116 cells. A colony formation assay (**l**) was used to detect the clonogenesis of the cells. Migration assay (**m**) was used to detect migration of the cells. **n** Quantitative real-time PCR analysis of *SMARCA4, EGFR,* and *TNS4* mRNA levels normalized to *GAPDH* in SMARCA4-WT, -R1157W HE and -R1157 HO HCT116 cells. $^{*}P < 0.05$, $^{**}P < 0.01$. **o** Western blot analysis of the indicated proteins in SMARCA4-WT, -R1157W HE and -R1157 HO HCT116 cells. GAPDH served as a loading control. In the graphs, the error bars are the standard deviation of the mean.

a

b

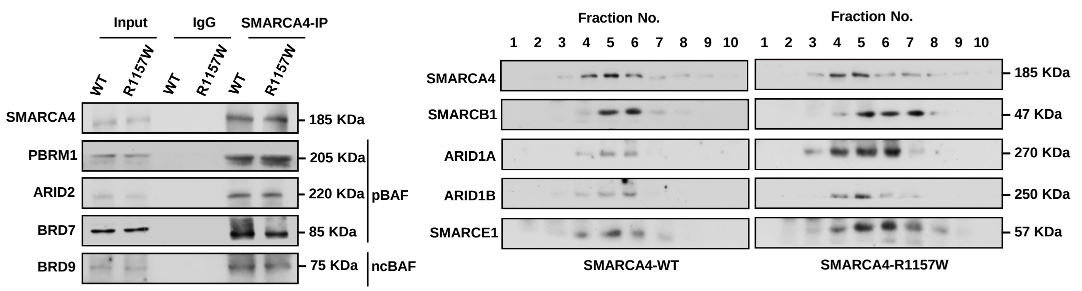

c

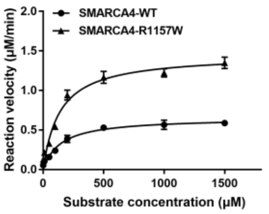

d

	SMARCA4-WT	SMARCA4-R1157W
V_{max} (μM min^{-1} mg^{-1})	108.8±3.66	241.6±8.16
K_m (μM)	144±18.29	138.3±17.41
K_{cat} (min^{-1})	0.065±0.002	0.145±0.004
K_{cat}/ K_m (min^{-1} μM^{-1})	0.453	1.048

Supplementary Figure 5. The SMARCA4-R1157W mutation enhances the stability of the cBAF complex and ATPase activity. **a** Coimmunoprecipitation assay with lysates prepared from HCT116 cells transfected with SMARCA4-WT or SMARCA4-R1157W plasmids. Anti-SMARCA4 antibody was used for the immunoprecipitation and lysates were immunoblotted for the indicated proteins. **b** Glycerol gradient sedimentation was performed to investigate the changes in the core subunit composition of the cBAF complex containing SMARCA4-WT or -R1157W mutant. FLAG immunoprecipitation of cytoplasmic extracts of SMARCA4-WT and -R1157W mutant HCT116 cells was separated by glycerol gradient sedimentation. SMARCA4-containing fractions were analyzed by western blotting using the indicated antibodies. **c-d** A kinetic assay was performed to measure the kinetic parameters of SMARCA4-WT and –R1157W mutant proteins on ATP. Reaction rates were determined by quantitating the fraction of ATP hydrolyzed at multiple time

points (**c**). Kinetic parameters were determined by fitting the initial rate data to the Michaelis-Menten equation using the Origin software (**d**). All reactions were performed in triplicate. In the graphs, the error bars are the standard deviation of the mean.

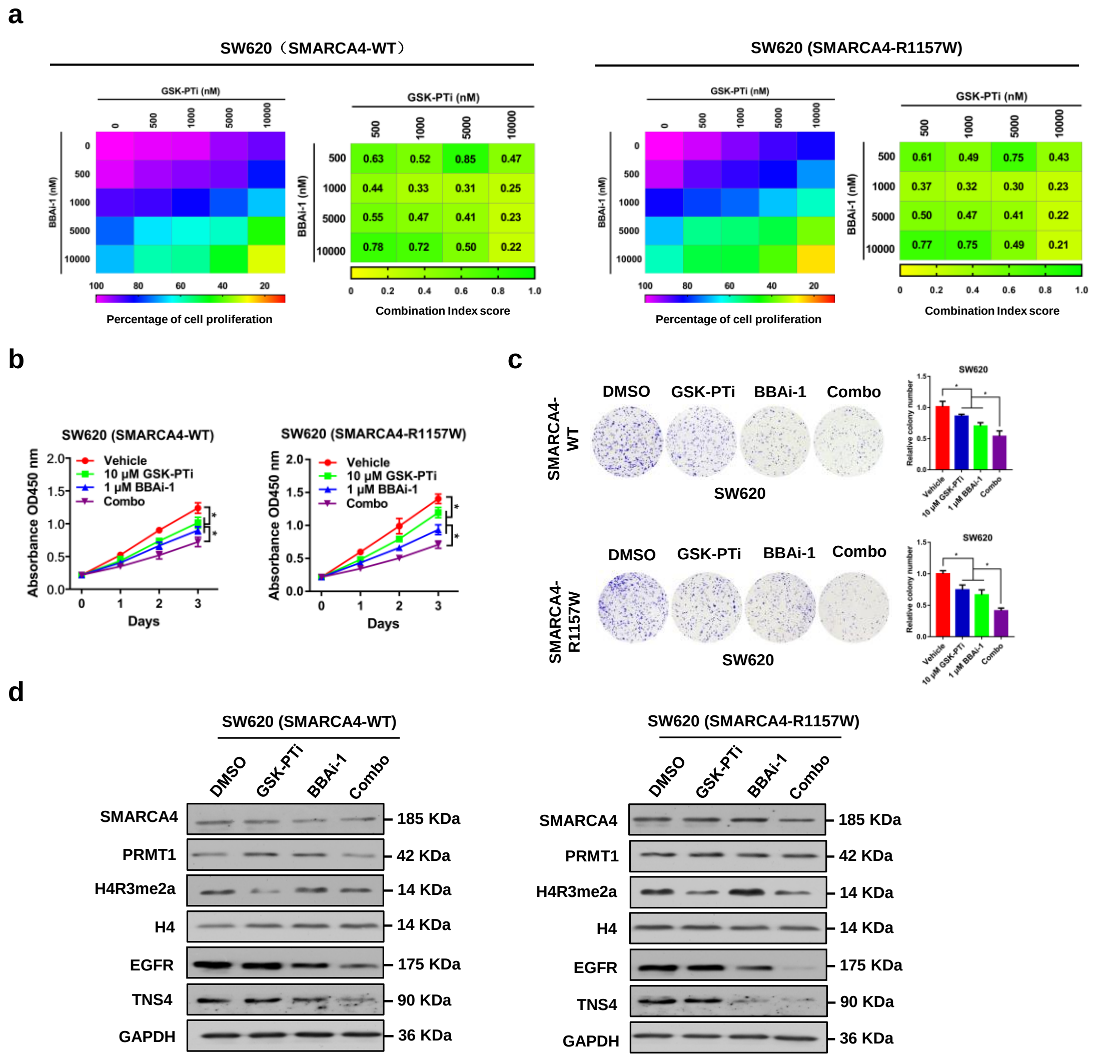

Supplementary Figure 6. Synergistic effect of combined treatment with GSK-PTi and BBAi-1 in SMARCA4-R1157W SW620 cells. a Drug dose-response matrix for growth inhibition of SMARCA4-WT (left panels) or -R1157W (right panels) SW620 cells following 7-day culture with GSK-PTi plus BBAi-1. Color gradation indicates percent viability at the indicated dose combination. CI (combination index) scores for SMARCA4-WT (left panels) or SMARCA4-R1157W (right panels) SW620 cells following 7 days of culture with GSK-PTi plus BBAi-1 at the indicated concentrations. Each CI score represents data from at least three independent experiments. **b-c** CCK8 (**b**) and colony formation (**c**) assay were performed to

evaluate the effect of the combination treatment with 10 μM GSK-PTi or/and 1 μM BBAi-1 on cell proliferation of SMARCA4-WT or -R1157W SW620 cells. *P < 0.05. **d** Western blot analysis of indicated proteins in SMARCA4-WT or -R1157W SW620 cells following treatment with 10 μM GSK-PTi or/and 1 μM BBAi-1. Histone H4 and GAPDH were used as loading control. In the graphs, the error bars are the standard deviation of the mean. In the graphs, the error bars are the standard deviation of the mean.

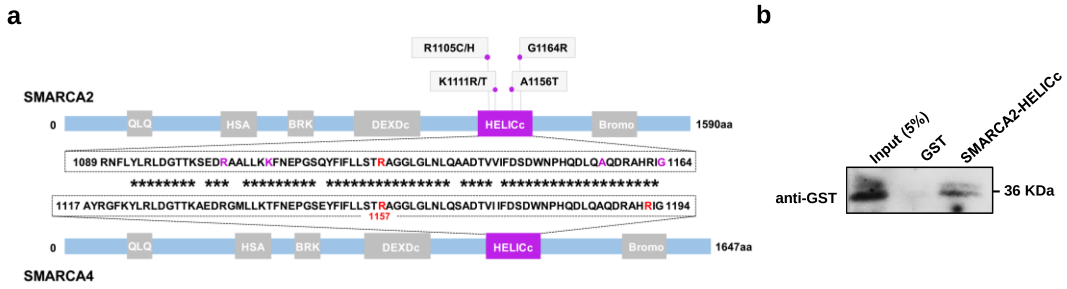

Supplementary Figure 7. The binding of SMARCA2 HELICc domain to H4R3me2a. a Comparison of the amino acid sequences of SMARCA2 and SMARCA4 HELICc domain and their mutations in CRC. **b** Peptide pulldown assays to detect the interaction between H4R3me2a peptide and SMARCA2 HELICc domain protein as a GST fusion protein. GST was used as a negative control.

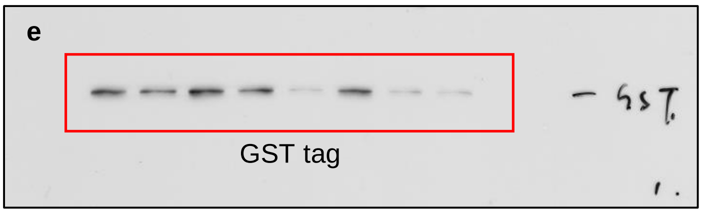

Supplementary Figure 8. Uncropped and unprocessed scans of immunoblots in Figure 2e.

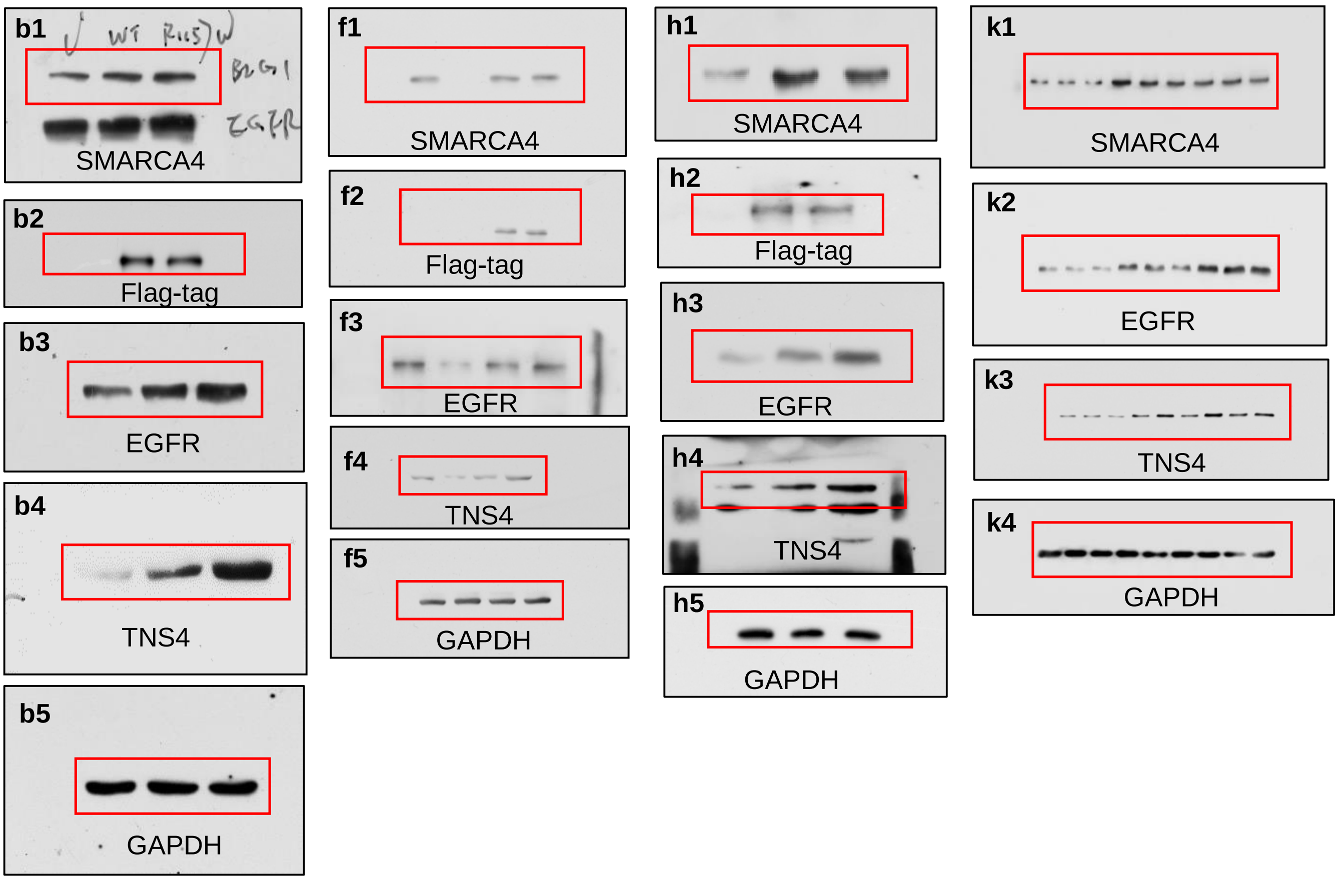

Supplementary Figure 9. Uncropped and unprocessed scans of immunoblots in Figure 3.

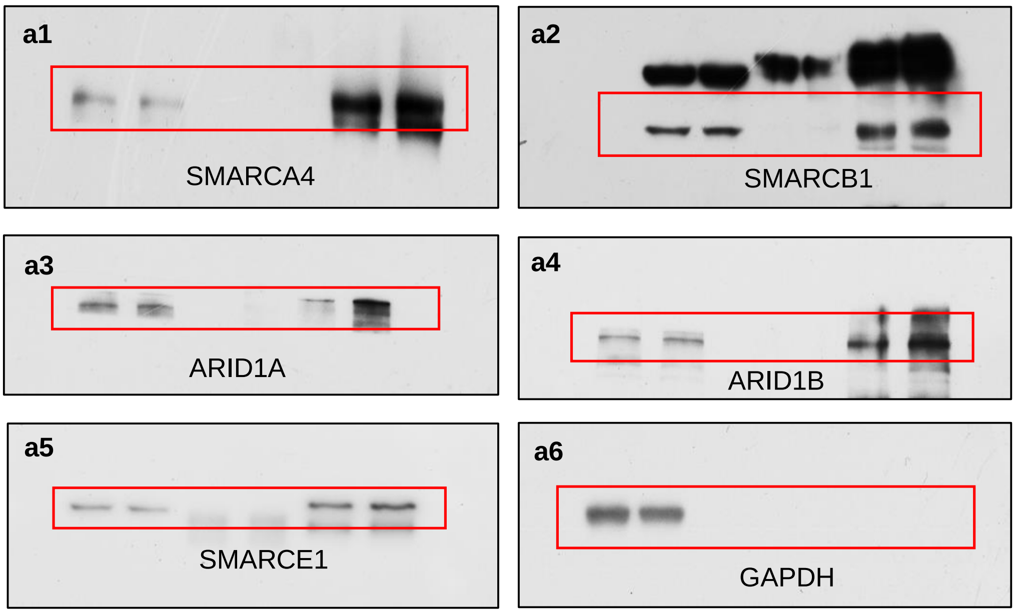

Supplementary Figure 10. Uncropped and unprocessed scans of immunoblots in Figure 4.

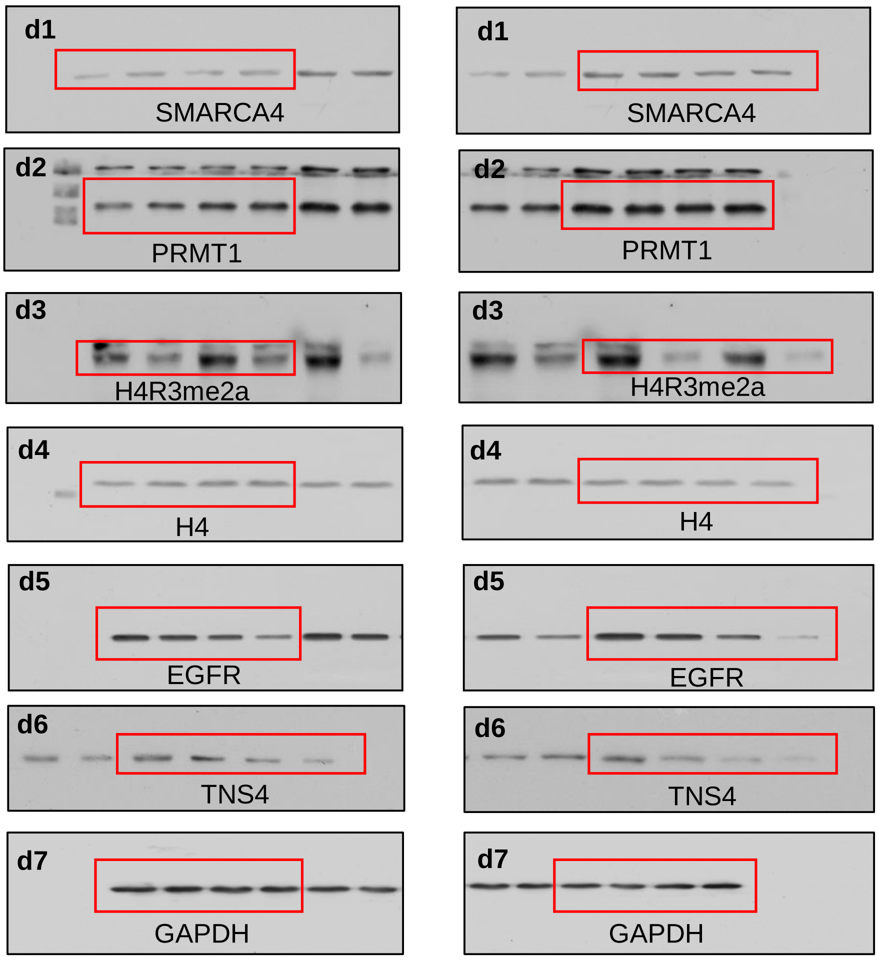

Supplementary Figure 11. Uncropped and unprocessed scans of immunoblots in Figure 5.

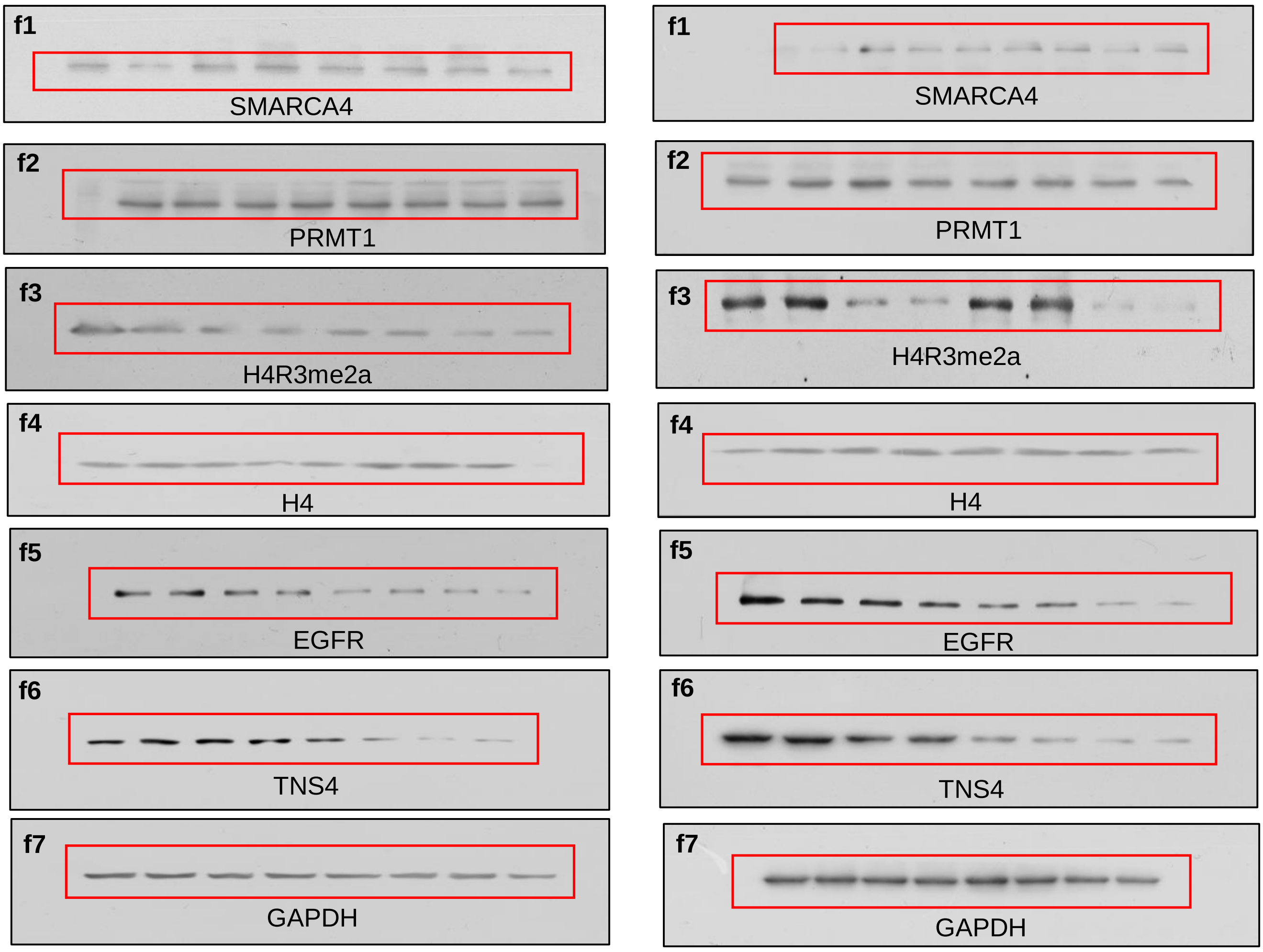

Supplementary Figure 12. Uncropped and unprocessed scans of immunoblots in Figure 6.

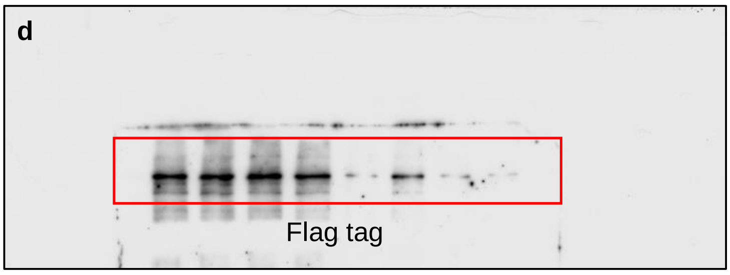

Supplementary Figure 13. Uncropped and unprocessed scans of immunoblots in Supplementary Figure 2.

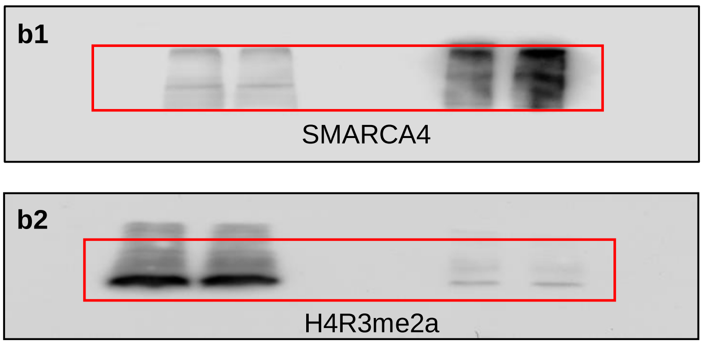

Supplementary Figure 14. Uncropped and unprocessed scans of immunoblots in Supplementary Figure 3.

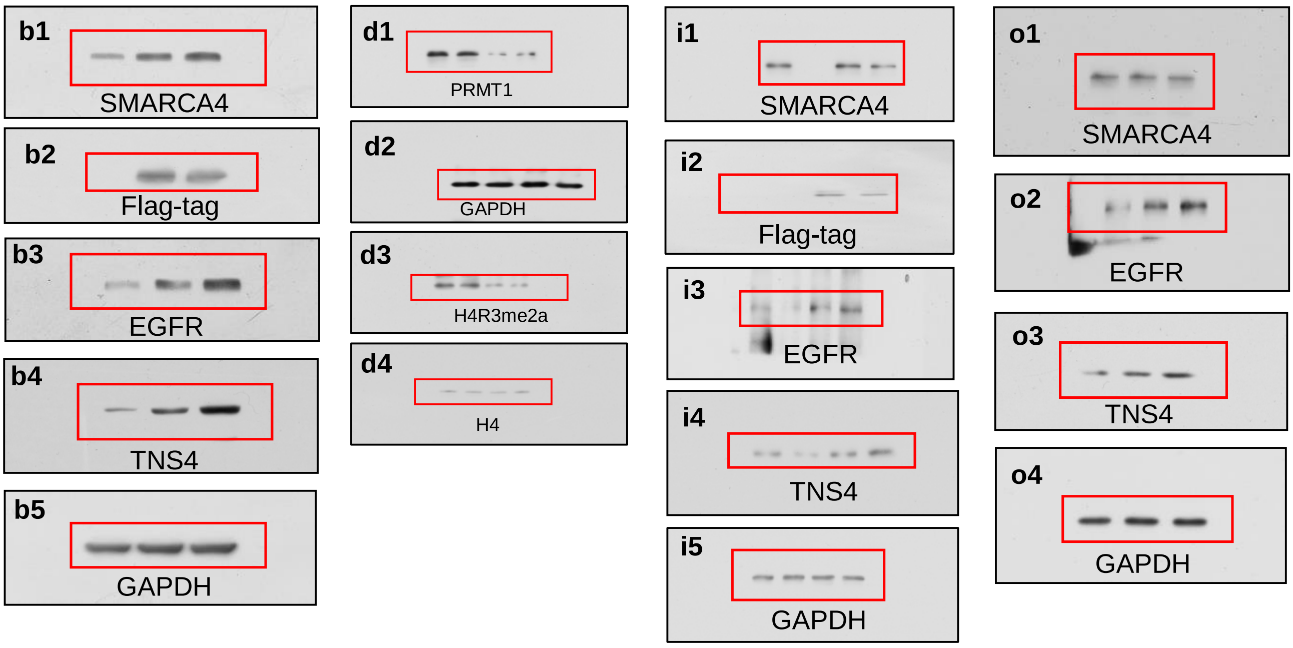

Supplementary Figure 15. Uncropped and unprocessed scans of immunoblots in Supplementary Figure 4.

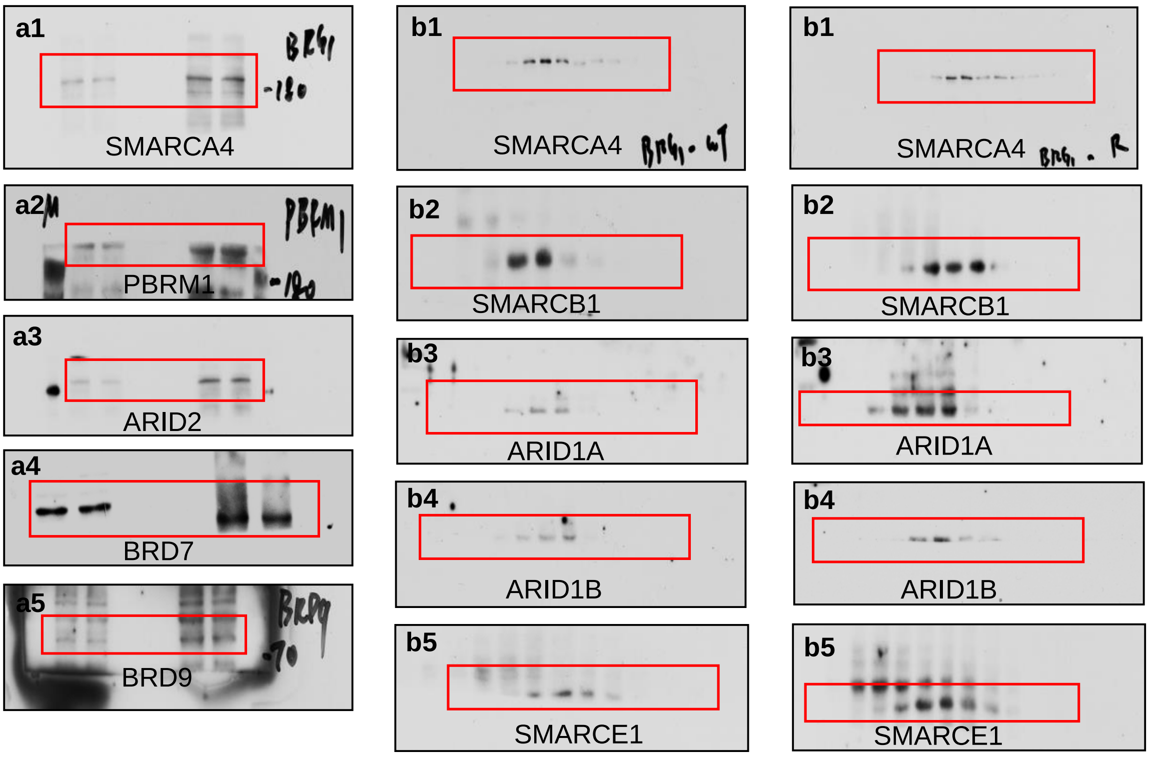

Supplementary Figure 16. Uncropped and unprocessed scans of immunoblots in Supplementary Figure 5.

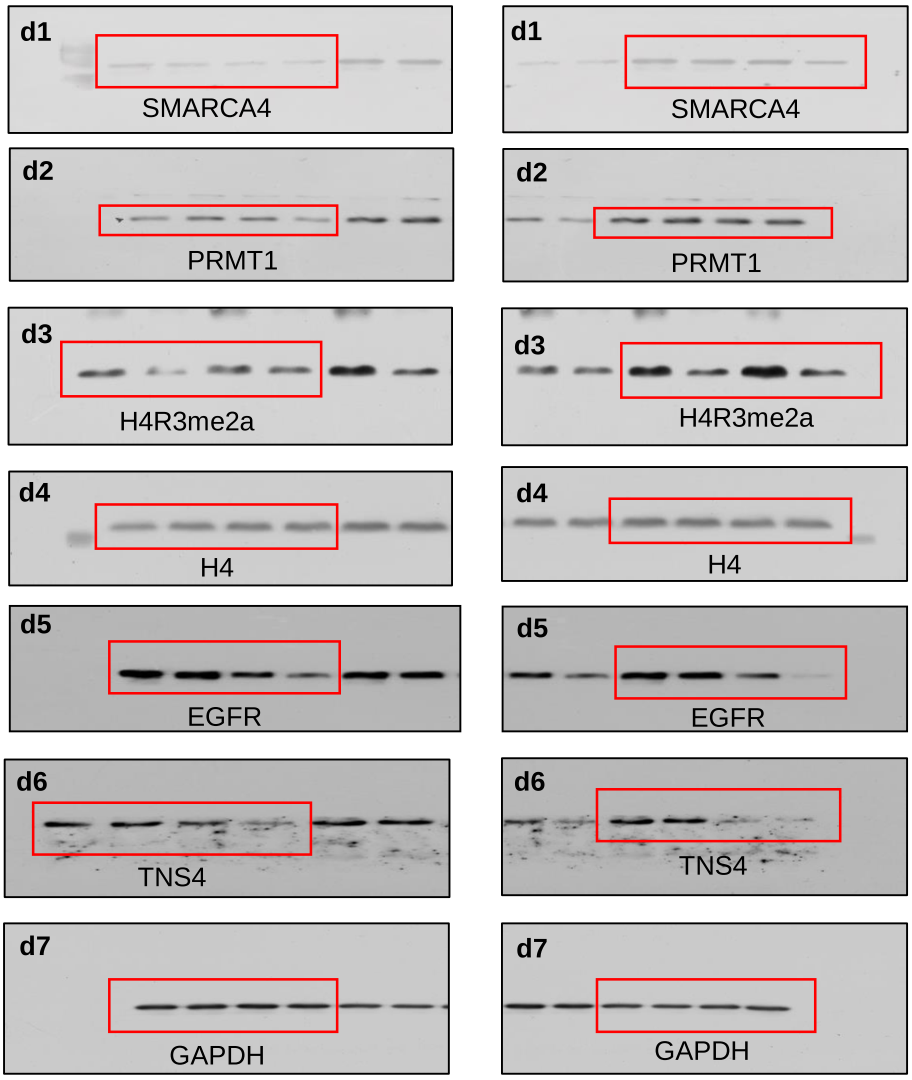

Supplementary Figure 17. Uncropped and unprocessed scans of immunoblots in Supplementary Figure 6.

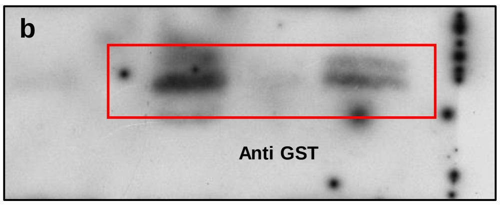

Supplementary Figure 18. Uncropped and unprocessed scans of immunoblots in Supplementary Figure 7.
